# Dynamic transcriptome profiling provides insights into rhizome enlargement in ginger (*Zingiber officinale Rosc*.)

**DOI:** 10.1371/journal.pone.0287969

**Published:** 2023-07-14

**Authors:** Yun Ren, Wen Bo Li, Zhe Xin Li, Wen Lin Zhang, Deng Wei Jue, Hai Tao Xing, Hong Lei Li, Qiang Li

**Affiliations:** 1 Chongqing Key Laboratory of Economic Plant Biotechnology, Collaborative Innovation Center of Special Plant Industry in Chongqing, Institute of Special Plants, Chongqing University of Arts and Sciences, Yongchuan, Chongqing, China; 2 School of Advanced Agriculture and Bioengineering, Yangtze Normal University, Fuling, Chongqing, China; China National Rice Research Institute, CHINA

## Abstract

The rhizome is an economically important part of ginger (*Zingiber officinale* Rosc.). However, the mechanism of ginger rhizome enlargement remains unclear. In this study, we performed an integrated analysis of the hormone content and transcriptome of ginger at three rhizome enlargement stages: initial enlargement (S1), middle enlargement (S2), and peak enlargement (S3). With rhizome enlargement, the levels of the hormones zeatin (ZT), gibberellic acid (GA), indole acetic acid (IAA), and jasmonic acid (JA) were significantly increased, and this increase was positively correlated with rhizome diameter. Transcriptomic analysis identified a large number of differentially expressed genes (DEGs); the number of DEGs were 2,206 in the transition from S1 to S2, and 1,151 in the transition from S2 to S3. The expression of several genes related to hormone biosynthesis and signalling and cell division or expansion, and transcription factors was significantly altered, which suggests that these genes play essential roles in rhizome enlargement. The results of correlation analysis suggested that the process of ginger rhizome enlargement may be primarily related to the regulation of endogenous cytokinin, GA_3_, auxin, and JA biosynthesis pathways and signal transduction; *GRAS*, *HB*, *MYB*, *MYB122*, *bZIP60*, *ARF1*, *ARF2*, *E2FB1*, and *E2FB2*, which may regulate the expression of rhizome formation-related genes; and *CYC2*, *CDKB1*, *CDKB2*, *EXPA1*, and *XTH7*, which may mediate cell division and expansion. These results provide gene resources and information that will be useful for the molecular breeding in ginger.

## Introduction

Modified roots or stems are economically important parts of many vegetable crops [1]. Clarifying the formation of modified roots or stems is an important research aim in plant developmental biology, and the outcomes of these researches can provide valuable theoretical basis for improving the yield and quality of vegetable crops [2]. Nevertheless, the process of rhizome development in ginger (*Zingiber officinale* Rosc.) remains unclear.

Plant hormones are the basic regulatory factors involved in the development of modified roots or stems [2]. Gibberellin acid (GA), cytokinin, and salicylic acid (SA) are found in high levels in the modified roots or stems of turnips, radishes, and lily bulbs, which are closely related to tuber expansion [3–5].

Furthermore, the interactions between hormones induce tuber initiation and growth; for example, the crosstalk between GA and auxins and between auxins and strigolactone (SL) in the stolon contributes to the overall potato tuber yield [6, 7]. Hormone-related genes are also involved in the development of modified roots or stems; for example, the abscisic acid (ABA) signalling pathway gene *ABR1* is involved in the expansion of tuberous stems in mustard tuber [8]. Moreover, the auxin-related genes *StPIN*-like and *acrA*-like, the jasmonic acid (JA) carboxyl methyltransferase gene *AtJMT*, and the SL biosynthesis pathway gene *CCD8* have been shown to be involved in the growth and enlargement of potato tubers [9–11].

Transcription factors (TFs) regulate the development of modified roots and stems [12, 13]. MADS-box TF genes have been shown to be involved in tuberization and are known to accumulate in the fast-growing stems of potatoes and during the early stages of potato enlargement [14]. *ABF4* promotes tuber formation by regulating gene expression in ABA and GA metabolism during tuber formation [15]. Homeobox (HB) TFs, such as *Ibkn1/2*, *StBEL5*, and *POTH1*, have been shown to interact physically and function as key molecular signals controlling storage organ development in five storage root crops: sweet potato, cassava, carrot, radish, and sugar beet [16, 17].

Cell division and cell expansion-related genes, including cyclin (*CYC*), cyclin-dependent protein kinase (*CDK*), RB/E2F TFs, expansin (*EXP*), and xyloglucan endotransglucosylase/hydrolase (*XTH*), play important roles in the expansion of fleshy rhizomes [18, 19]. *CycD3* regulates the cell division and differentiation in fleshy roots of radish [20]. Two types of CDK genes (*CDKA* and *CDKB*) and three types of E2F TFs (*E2Fa*, *E2Fb*, and *E2Fc*) were found to be involved in the cell cycle regulation of mustard tuber enlargement and play an important role in cell division in mustard tuber [21]. Sweet potato *IbEXP1* has been reported to play a negative role in the formation of storage roots by suppressing the proliferation of metaxylem and cambium cells and thus inhibiting the initial thickening of storage roots [22]. *BjXTH1* and *BjXTH2* are specifically expressed in cells that are highly re-replicated in the nucleus of the pith during the expansion of the tuberous stem of mustard [21].

Ginger is one of the most economically valuable medicinal and edible plants in the Zingiberaceae family, and the rhizome is its main product [23]. However, the regulatory mechanisms that control rhizome enlargement remain unclear. Recently, studies based on RNA-seq analysis revealed gingerol biosynthesis, cellulose production, and the response to post-harvest dehydration stress in ginger [24–27]. Accordingly, we used RNA-seq technology to investigate the changes during the three enlargement stages of the ginger rhizome and analysed the possible regulatory mechanisms of rhizome enlargement taking into account plant hormones, TFs, the cell cycle, and cell expansion.

## Materials and methods

### Plant materials

**Fig 1:**
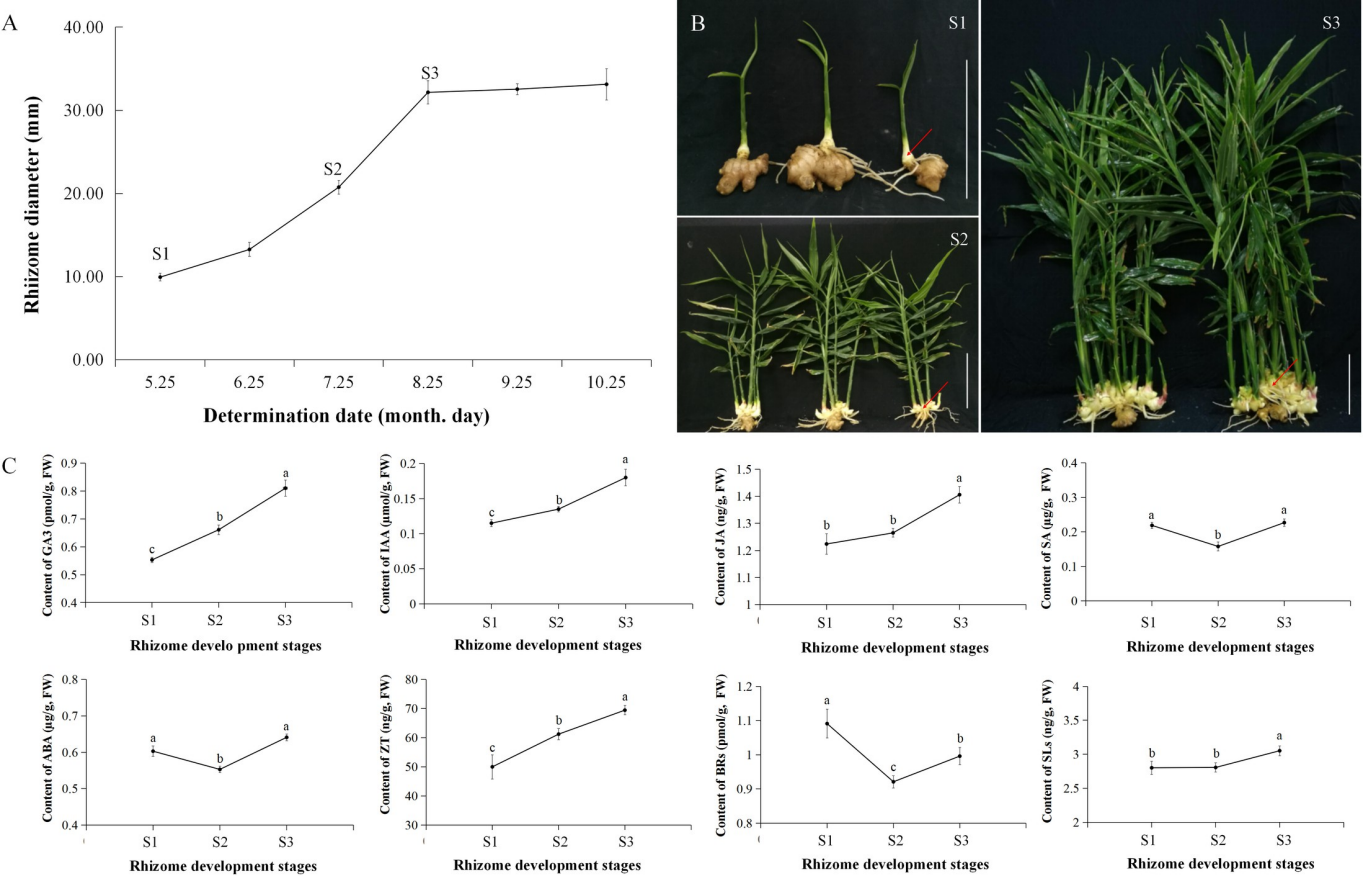
Rhizome enlargement process and contents of endogenous hormones during ginger rhizome enlargement. A and B. Rhizome enlargement process and phenotype of three different stages, including stage 1 (S1), the initial enlargement stage; stage 2 (S2), the middle enlargement stage; and S3, the peak enlargement stage of rhizome development. Bar = 15 cm; The red arrow is the graphical representation of samples. C. Representative hormone production at the three different stages. Three biological replicates of every enlargement stage were performed. Values represent the average ± SD. Different letters indicate significant difference at *P* = 0.05 level.

The plants of 57 ginger varieties (S1 Table) were grown in a greenhouse at the Chongqing University of Arts and Sciences, Chongqing, China. The three enlargement stages (initial enlargement [S1], middle enlargement [S2], and peak enlargement [S3]) of *Zingiber officinale* Roscoe. ‘RY20’ were designated according to the enlargement of the first main rhizome (Fig 1): new young main rhizome (S1), continued to expand for about 60 days to form the first and second lateral branches on the outside (S2), continued to expand for about 30 days to mature size accompanied by the formation of the third and fourth lateral branches (S3). At each stage, the first main branch of the rhizome was used for hormone determination and transcriptome sequencing. Each collection stage comprised three biological replicates, and each replicate consisted of tissues from three different plants. All samples were rinsed with sterile distilled water, immediately frozen in liquid nitrogen, and stored at –80 °C for hormone quantification and RNA isolation.

### RNA preparation and Illumina sequencing

RNA was isolated using TRIzol reagent (Invitrogen, USA), and cDNA libraries were prepared (cat#E7420; New England Biolabs, USA) from all samples according to the manufacturer’s instructions and Tanaka’s method [16]. Three replicates were sequenced separately for each stage. Library sequencing was performed on an Illumina HiSeq 2500 platform to obtain 150-bp paired-end reads. The filtered reads were corrected using the Rcorrector software to erase sequencing errors. After filtering and correction, the clean reads from all libraries were pooled and assembled into transcripts using Trinity version 2.2.0, with the default parameters. Transcripts shorter than 300 bp were discarded, and the longest transcript in each cluster was selected as the representative unigene. The transcriptomes were assembled using the de Bruijn method [28], and the longest transcript in each de Bruijn graph was defined as a unigene and used as the reference sequence for assembly and coding sequence (CDS) prediction. The CDS and protein sequences of the unigenes were predicted using TransDecoder (http://transdecoder.github.io/). Assembly sequences referred to as unigenes were annotated through a BLASTX/BLASTP similarity search using the Trinotate software. The functions of all the annotated unigenes were determined by Clusters of Orthologous Groups of proteins (COG), Gene Ontology (GO), and Kyoto Encyclopaedia of Genes and Genomes (KEGG) databases (E-value ≤ 1e^−5^) with the two published entire annotated genome as the reference set [29, 30].

Clean reads from all samples were pooled and the read counts were normalized to the aligned Fragments Per Kilobase of transcript per Million (FPKM) mapped reads to quantify the gene expression level using cufflinks (version: 2.1.1). Differentially expressed genes (DEGs) were identified using Cuffdiff software (version: 2.1.1). The significance of DEGs was determined using |Log2 FC≥1| as a cutoff. False discovery rate (FDR) was adjusted across genes for significance levels (≤0.05) of all tests.

To obtain knowledge about expression profiles of DEGs throughout three enlargement stages, the short time-series expression miner (STEM) was used to cluster DEGs. The gene expression data and gene annotation files were uploaded to STEM. Expression profiles were analyzed using the STEM clustering algorithm.

BiNGO 2.3 plugin tool in Cytoscape 3.2.1 was used to gain knowledge about gene ontology (GO) terms of DEGs in each cluster. Over-represented GO terms were identified using a hypergeometric test with a significance threshold of 0.05 after correction by Benjamini and Hochberg FDR with our recently published entire annotated genome as the reference set (Li et al., 2021).

Mapman visualization was performed as described previously to identify gene families that may play essential roles in rhizome enlargement. Contigs were classified into a set of hierarchical functional categories (BINs), using Mercator with a blast cutoff of 50. Because one unigene might have multiple contigs, a functional term of a unigene was derived from its representative contig that had the highest bit score. Enrichment analysis was completed through Fisher’s test using Mefisto (http://www.usadellab.org/cms/index.phppage=mefisto) with Bonferroni correction. Gene expression changes were viewed in Mapman 3.6.0RC1.

### LC-MS/MS analysis of endogenous phytohormones content

A liquid chromatography-tandem mass spectrometer (LC-MS/MS, Shim-pack UFLC SHIMADZU CBM30A system; MS, Applied Biosystems 6500 Q TRAP, USA), equipped with an electrospray ionisation source, was used to identify phytohormones and quantify their content in the samples [31]. The ginger rhizomes collected above were used for profile analysis of endogenous phytohormones, including GA, indole acetic acid (IAA), zeatin (ZT), JA, ABA, SA, brassinosteroids (BRs), and SLs.

0.1 g of tissues was ground into a fine powder with a mortar and pestle in liquid nitrogen and extracted with 1 mL of ice-cold extraction buffer (isopropanol-hydrochloric acid solvent) for 30 min at 4 °C. 2 mL of dichloromethane was added and the mixture was incubated at 4 °C for 30 min with shaking. The extractant was separated by centrifugation at 12,000 g for 5 min at 4 °C. Then the organic phase was collected, dried under nitrogen and dissolved in 400 μl of methanol (in 0.1% formic acid, v/v). Finally, the concentrated solutions passed through 0.22 μm filter before determination by liquid chromatography-electrospray ionization-mass spectrometry (LC-ESI-MS). HPLC-MS/MS analysis was carried out on a Shim-pack UFLC SHIMADZU CBM30A system (Shim-pack, Japan) coupled with an AB SCIEX QTRAP®6500 mass spectrometer (Applied Biosystems, USA). A poroshell 120 SB-C18 column (2.1 mm × 150 mm i.d., 2.7 μm) was used for separation. All compounds were base line separated using a binary solvent gradient (Solvent A: 0.1% formic acid in methanol; Solvent B: 0.1% formic acid in water). A linear solvent gradient program was as follows: 0.0–1.0 min: 20% A; 1.0–9.0 min, linear gradient from A/B (20:80) to A/B (80/20); 9.0–10.0 min: 80% A; 10.0–10.1 m in. linear gradient from A/B (80:20) to A/B (20/80); 10.1–15.0 min: 20% A. The injector volume selected was 2 μL. The ESI interface in the positive and negative MRM mode was chosen for the identification and quantification of the compounds. The set of parameters used is shown as follow: ion source spray voltage (IS), 4500 V; atomization temperature (TEM), 400°C; atomization gas pressure (GAS1), 65 Psi (nitrogen); heated auxiliary gas (GAS2), 70 Psi (nitrogen); air curtain gas pressure (CUR), 15 Psi (nitrogen). Quantification of hormones was carried out using a multiple reaction monitoring (MRM) method. For each stage, three biological replicates were performed. The specific diagnostic ions for each of the plant hormones are listed in S2 Table.

### Quantitative polymerase chain reaction (qPCR) analysis

qPCR was conducted to validate the expression levels of 25 randomly selected potential regulatory DEGs. One microgram of RNA, treated with gDNA-remover to eliminate genomic DNA, was reverse transcribed using an oligo(dT) primer according to the manufacturer’s protocol (HiScript III 1st Strand cDNA Synthesis Kit; Vazyme, China). qPCR analysis was performed with the ChamQ SYBR Green master mix (Vazyme, China) using LightCycler 96 (Jena, Germany) under the following cycling conditions: pre-denaturation at 95 °C for 30 s, followed 40 cycles at 95 °C for 5 s and 60 °C for 30 s. Each treatment group had three biological replicates, and the experiment was performed in triplicate. Melting curve analysis was performed, and the absence of non-specific products and primer dimers was verified. Relative expression levels were calculated using the 2^–△△Ct^ method. Expression levels of the target genes were normalised to those of the internal control gene, *Actin* [27]. The genes and primers used are shown in S3 Table.

### Correlation analysis

Pearson’s correlation coefficients were calculated for hormone content and rhizome diameter (RD). For this, the mean hormone content and the mean RDs of each enlargement stage of *Zingiber officinale* ‘RY20’ were calculated. The coefficients were calculated via multivariate and correlation analyses using the DPS data processing system (v15.10). The parameters with |*r*|≥ 0.9 were considered to be significantly correlated.

Pearson’s correlation coefficients were calculated for hormone contents/RDs and transcriptome data integration. The mean hormone content/RD at each stage and the mean expression levels of each transcript in the transcriptome data were calculated. The fold changes in each stage were then calculated in both the hormone and transcriptome data and compared to those in the previous stage. Finally, the coefficients were calculated from the log2 (fold change) value of each hormone/RD and transcript using DPS (v15.10). Correlation coefficients of *r*^2^ > 0.9 were selected. The hormone content/RD and transcriptome relationships were visualized using Cytoscape (version 3.7.2).

Pearson’s correlation coefficients were calculated for the RD of 15 different ginger varieties (listed in bold type in S1 Table and S1 Fig) and the expression levels of 30 correlated genes. The mean RDs of all biological replicates of each ginger variety and the mean expression level of each DEG qPCR result were calculated. Finally, the coefficients were calculated using the DPS data processing system (v15.10) with multivariate and correlation analyses. Correlation coefficients corresponding to *r* ≥ 0.6 were considered significant (*r* ≥ 0.8 was considered, very strong correlation, and 0.6 ≤ *r* < 0.8 was considered a strong correlation). Rhizomes of the first main branch at S3 were collected to measure RD and conduct qPCR. Each ginger variety was sampled from three biological replicates.

### Data curation and statistical analysis

The measured data were sorted by Excel 2010, and SPSS21.0 software was used for statistical analysis of the sorted data. The Min-Max method was used to standardize the relative expression data of each gene, and the transformation formula: (n-minimum value)/(maximum value-minimum value) was used to make all trait values in a specific interval of 0–1, where n is an independent variable. Finally, the heat map is drawn by TBtools software.

## Results

### Rhizome enlargement process and quantitative content of endogenous hormones in rhizomes at different enlargement stages

The dynamics of RD of the first main branch of the ginger ‘RY20’ plants are shown in Fig 1A; from the seedling stage on the May 25th to the enlargement stage on the August 25th, RD increased rapidly and then entered a growth plateau period, in which there were no appreciable changes in RD. The rhizomes that were collected on the May 25th, July 25th, and August 25th were marked as S1 (RD = 9.93 ± 0.46 mm), S2 (RD = 20.75 ± 0.85 mm), and S3 (RD = 32.16 ± 1.41 mm), respectively (Fig 1B). Endogenous hormone production was measured during the three rhizome enlargement stages (Fig 1C). Significant increases in gibberellic acid (GA_3_), IAA, and ZT were detected during stages S1 to S3. The content levels of ABA, SA and BRs decreased significantly from S1 to S2, and increased significantly from S2 to S3. JA content increased slightly during S1-S2 and increased significant during S2-S3, while SLs decreased slightly around S2 and then increased significant to S3.

### Dynamic transcriptome profiles during rhizome enlargement

**Fig 2:**
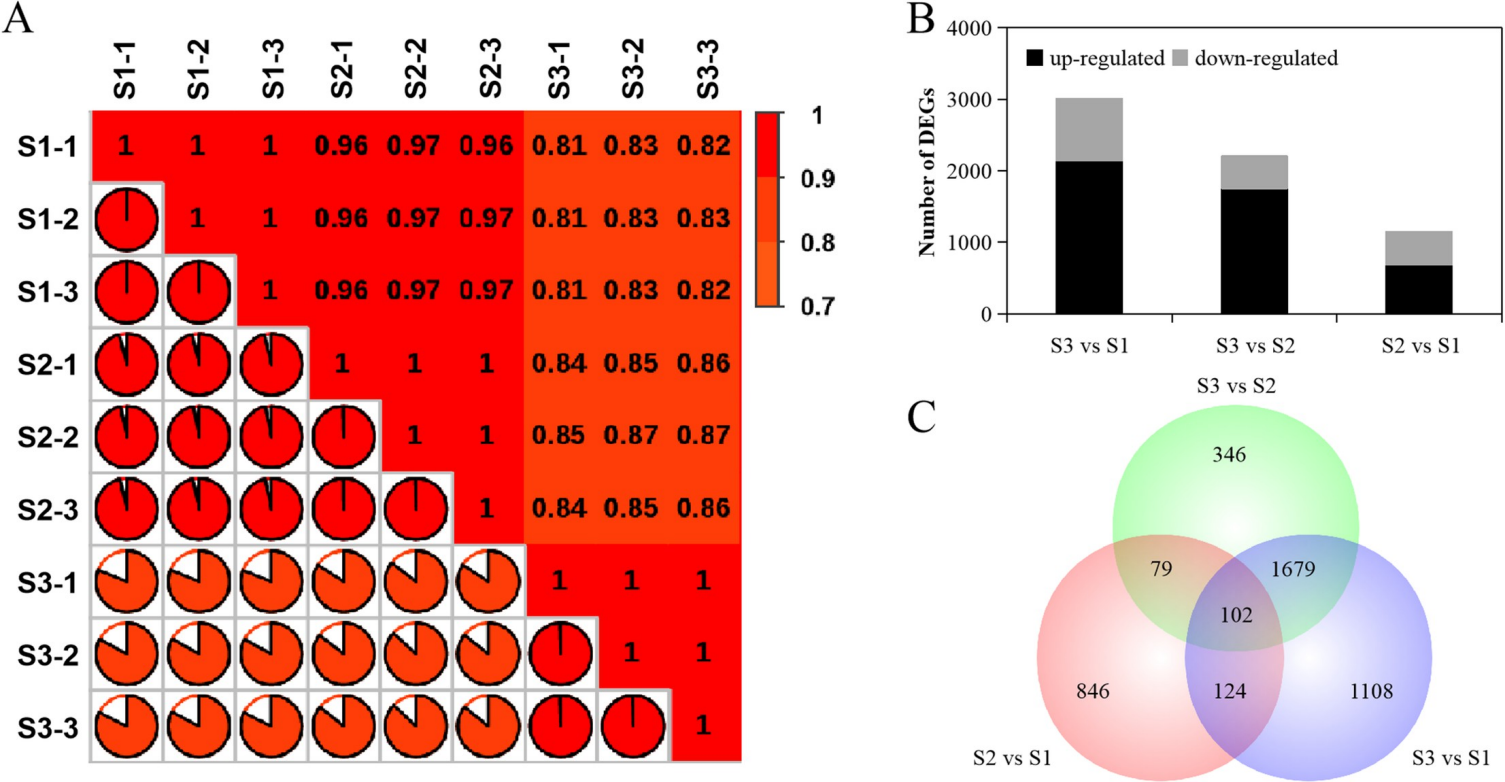
Cluster heatmap and numbers of potential differential unigenes in different development stages of ginger. A) Cluster heatmap, the different colours are the *r*^2^ values of the Pearson’s correlation coefficient; red represents high correlation, and yellow represents low correlation. B) Number of differentially expressed genes (DEGs) in ginger rhizomes between stage 2 (S2) and S1, between S3 and S2, and between S3 and S1; black represents significantly upregulated genes, grey represents significantly downregulated genes. C) Venn diagram of differential unigenes; potential differential unigene numbers between S2 and S1, between S3 and S2, and between S3 and S1.

RNA sequencing of the three-stage libraries resulted in 49,974,927–54,757,400 clean reads from S1 to S3, respectively, and the percentage of bases with a quality score of 30 (Q30) in each sample was not less than 93.42% (S4 Table). The mapping rate was over 76.96% for samples in each stage (S4 Table), and cluster heatmap results showed that three biological replicates were strongly correlated (*r*^2^ = 1) (Fig 2A), indicating that the sequencing results met the requirements of the subsequent experiments.

A total of 4,284 unigenes were significantly differentially expressed across these stages, and the number of DEGs were 3,013 between S1 and S3, 2,206 between S2 and S3, and 1,151 between S1 and S2 (Fig 2B and 2C). Of these genes, 2,141, 1,755, and 787 were upregulated and 872, 451, and 364 were downregulated in the transition periods S1-S3, S2-S3, and S1-S2, respectively (Fig 2B). A total of 102 DEGs shared similar expression patterns (Fig 2C), which may play a critical role in the regulation of rhizome enlargement in ginger.

**Fig 3:**
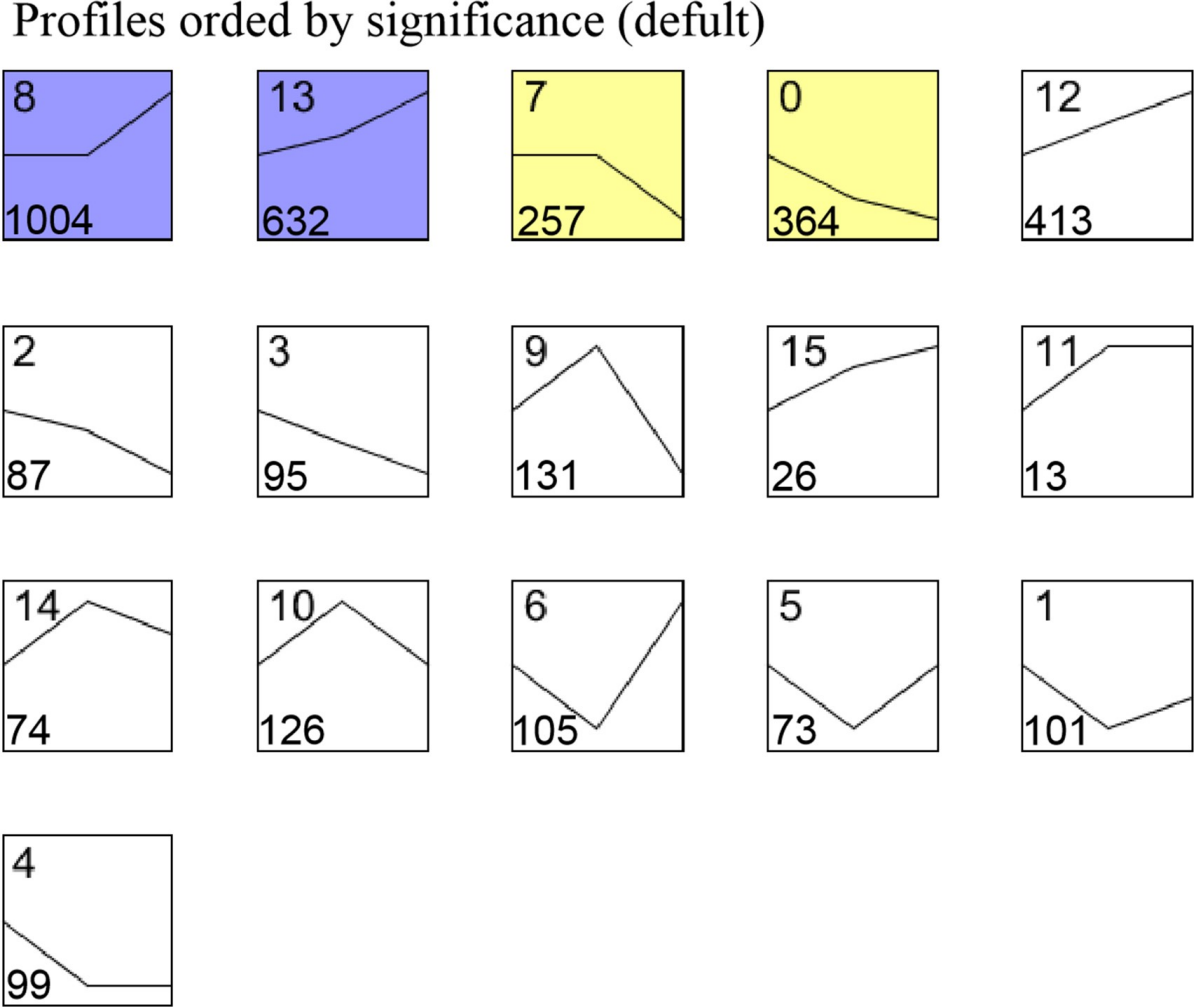
Cluster and STEM analysis of differentially expressed genes (DEGs). Sixteen clusters were obtained using the STEM software. The coloured clusters represent a significant level (*p* ≤ 0.05). The number at the top is the cluster number. The number at the bottom is the gene number assigned to each cluster.

Cluster analysis of the DEGs by STEM generated 16 clusters, including downregulated genes in clusters 0–7 and upregulated genes in clusters 8–15 (Fig 3). The downregulated clusters 0 and 7 and the upregulated clusters 8 and 13 were statistically significant (*P* ≤ 0.05), and the number of genes assigned to clusters 0, 7, 8, and 13 was 364, 257, 1,004, and 632, respectively (Fig 3).

### Identification of up- and downregulated GO terms at distinct time points

**Fig 4:**
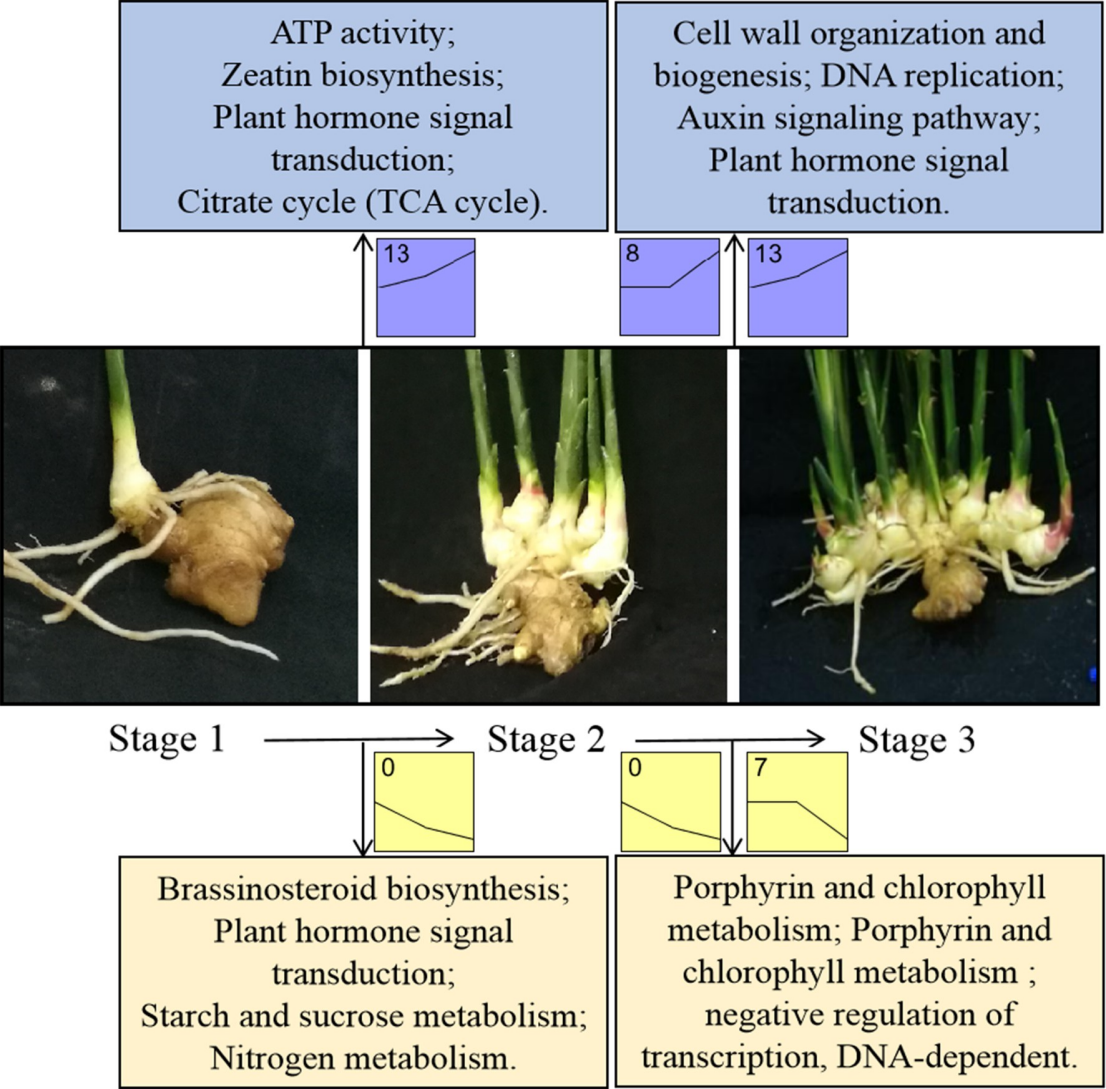
Metabolic processes and cellular component activated or repressed at different time points during ginger rhizome enlargement. The overrepresented Gene Ontology (GO) terms for the combined clusters of genes either upregulated (clusters 8 and 13) or downregulated (clusters 0 and 7) during rhizome enlargement were enriched using BiNGO [28, 30]. Significantly upregulated or downregulated GO terms at each time point during rhizome enlargement are indicated.

GO enrichment analysis of DEGs (in clusters 0, 7, 8 and 13) showed that the expression of genes involved in the ‘Starch and sucrose metabolism’, ‘Nitrogen metabolism’, ‘Brassinosteroid biosynthesis’ and ‘Plant hormone signal transduction’ were downregulated at the transition from S1 to S2 (cluster 0, Fig 4), whereas ‘ATP activity’, ‘Zeatin biosynthesis’, ‘Plant hormone signal transduction’, and ‘Citrate cycle (TCA cycle)’ were upregulated (cluster 13, Fig 4). During the transition from S2 to S3, the expression of genes involved in ‘Cell wall organization and biogenesis’, ‘DNA replication’, ‘Auxin signalling pathway’ and ‘Plant hormone signal transduction’ was significantly upregulated (clusters 8 and 13, Fig 4), whereas the expression of genes involved in ‘Porphyrin and chlorophyll metabolism’ and ‘Negative regulation of transcription, DNA-dependent’ was downregulated (clusters 0 and 7, Fig 4).

### Differential gene expression in hormone biosynthesis and signalling pathways

**Fig 5:**
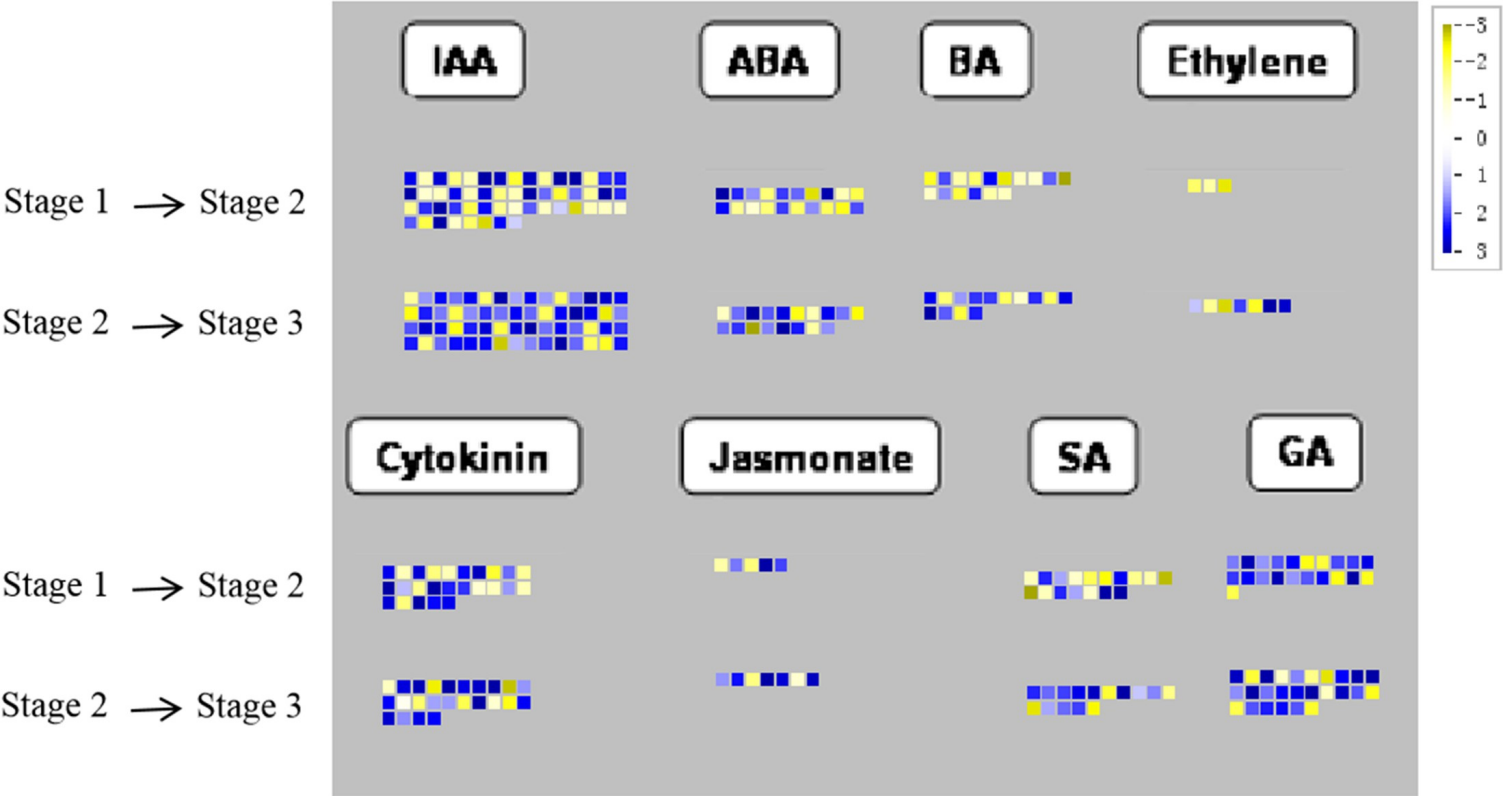
Display of gene expression involved in hormone biosynthesis and signalling pathway. Significantly differentially expressed genes (DEGs) (log2 fold change (FC) ≥ 1, FDR ≤ 0.05) were visualised using the Mapman software and organised into functional categories (BINs). Blue indicates a decrease and yellow indicates an increase in gene expression (see colour set scale in top right corner). IAA, auxin; ABA, abscisic acid; BA, brassinosteroid; SA, salicylic acid; GA, gibberellin. Detailed information on each gene and its expression level is listed in S5 Table.

Phytohormone-related DEGs (in clusters 0, 7, 8, and 13) were further analysed using the Mapman software. Among these, the largest number of DEGs were involved in auxin biosynthesis and signalling pathways, followed by the GA and cytokinin pathways (Fig 5 and S5 Table). DEGs related to auxin and cytokinin were predominantly upregulated through the S1–S2 (13/32 and 8/14) and S2–S3 (24/32 and 11/14) transitions, respectively (S5 Table). Among these DEGs, the expression of two SAUR-like genes, two auxin-induced genes, an auxin efflux carrier component, two cytokinin-N-glucosyltransferases, and three cytokinin receptors were upregulated through both the S1–S2 and S2–S3 transitions. Eight DEGs related to the GA pathway were upregulated in both transitions. Among these, *GA20ox*, *GA3ox*, and *GA13ox*, as well as GA-receptor, GA-responsive, and GA-regulated genes, were upregulated during rhizome enlargement; and GA2ox genes were also upregulated (S5 Table).

DEGs related to ABA and BR also accumulated through the S1–S2 (2/7 and 3/6) and S2–S3 (5/7 and 5/6) transitions, respectively (S5 Table). Only the expression of two ethylene (ET)-related DEGs was upregulated in S2–S3. Meanwhile, the expression of the three genes related to JA was upregulated through both transitions (S5 Table). Two and five DEGs related to SA were upregulated through the S1–S2 and S2–S3 transitions, respectively (S5 Table). In addition to these eight hormones, DEGs were identified in the SL pathway. Two SL esterase *DAD2s* were upregulated through the S1–S2 transition and the other two, *D14* and *RMS3*, were upregulated through the S2–S3 transition (S5 Table).

### Differential expression related to transcription factors

**Fig 6:**
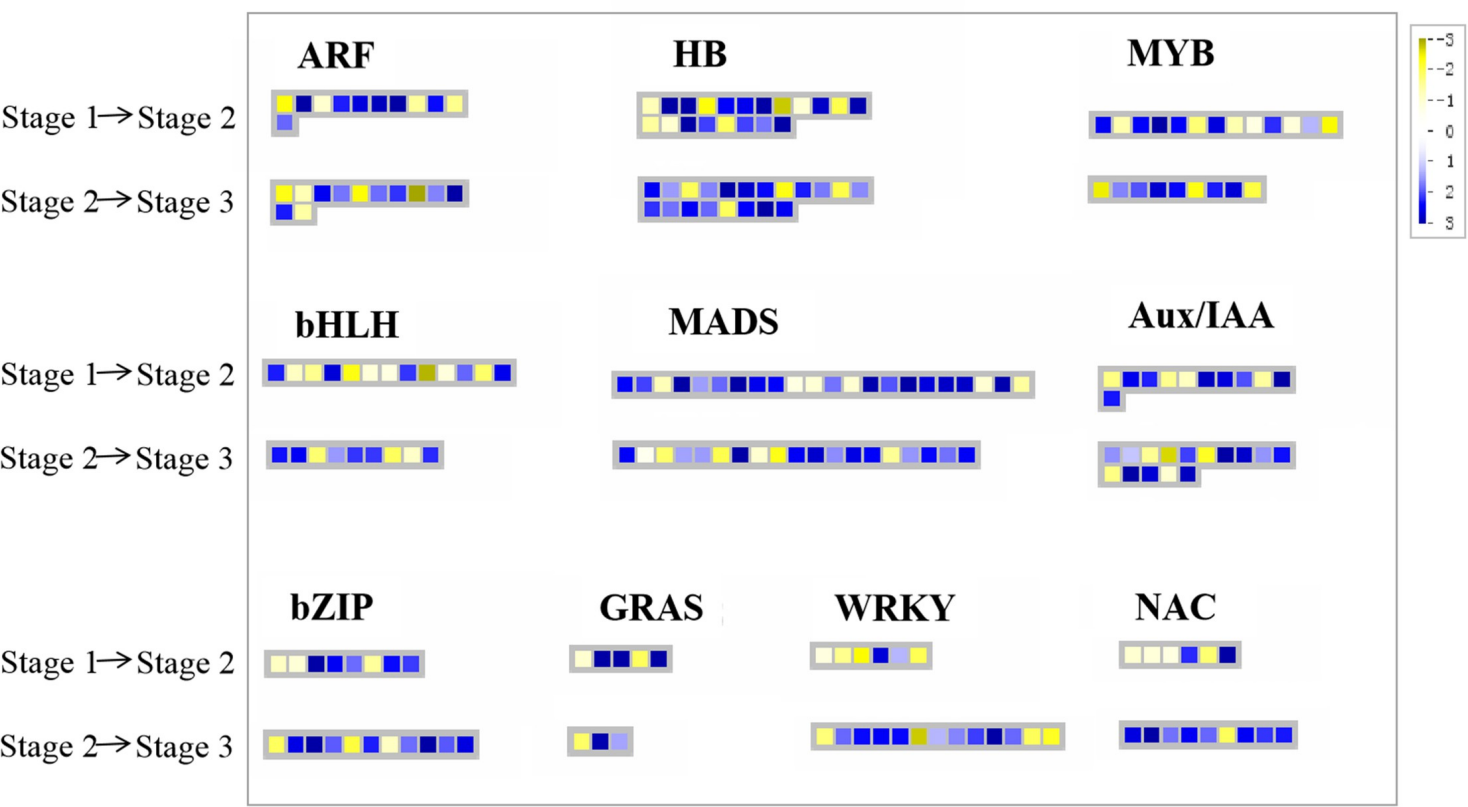
Display of gene expression of transcription factors (TFs). Significantly differentially expressed genes (DEGs) (log2 fold change (FC) ≥ 1, FDR ≤ 0.05) were visualised using the Mapman software and organised into functional categories (BINs). Blue indicates a decrease and yellow indicates an increase in gene expression (see colour set scale in top right corner). Detailed information on each gene and its expression level is listed in S6 Table.

Furthermore, TFs were analysed with Mapman and major TF families, including *ARF*, *HB*, *MYB*, *NAC*, *WRKY*, *MADS-box*, *bHLH*, *bZIP*, *GRAS*, and *Aux/IAA* families, were identified (Fig 6 and S6 Table). Among these TFs, *MADS-box* was the predominant family at the S1–S2 transition (with 24 DEGs including 15 upregulated genes), followed by the *HB* (11/21), *Aux/IAA* (7/13), *ARF* (7/9), and *MYB* (6/12) families (Fig 6 and S6 Table). *HB* was the predominant family at the S2–S3 transition (with 21 DEGs, including 14 upregulated genes), followed by the *MADS-box* (12/24), *Aux/IAA* (10/13), *MYB* (10/12), *WRKY* (9/9), and *NAC* (8/9) families (S6 Table). Among these TFs, three *MADS-box*, four *HB*, and four *Aux/IAA* family genes were upregulated through both the S1–S2 and S2–S3 transitions (S6 Table).

### Differential expression related to cell division and expansion

DEGs related to cell division and expansion across the S1–S2 and S2–S3 transitions were analysed. DEGs related to *CYCs* and *CDKs* were upregulated through both the S1–S2 (6/14 and 9/17) and S2–S3 (9/14 and 10/17) transitions (S7 Table). DEGs related to *EXPs* and *XTHs* were upregulated through both the S1–S2 (8/12 and 6/11) and S2–S3 (6/12 and 8/11) transitions (S7 Table). Only two *E2FB* TFs were differentially expressed, both of which were upregulated through the S1–S2 and S2–S3 transitions (S7 Table). Only one DEG related to *CYC* and two DEGs related to *CDKs*, *EXPs*, and *XTHs* were upregulated through both the S1–S2 and S2–S3 transitions (S7 Table).

### Verification of RNA-seq results with qPCR

**Fig 7:**
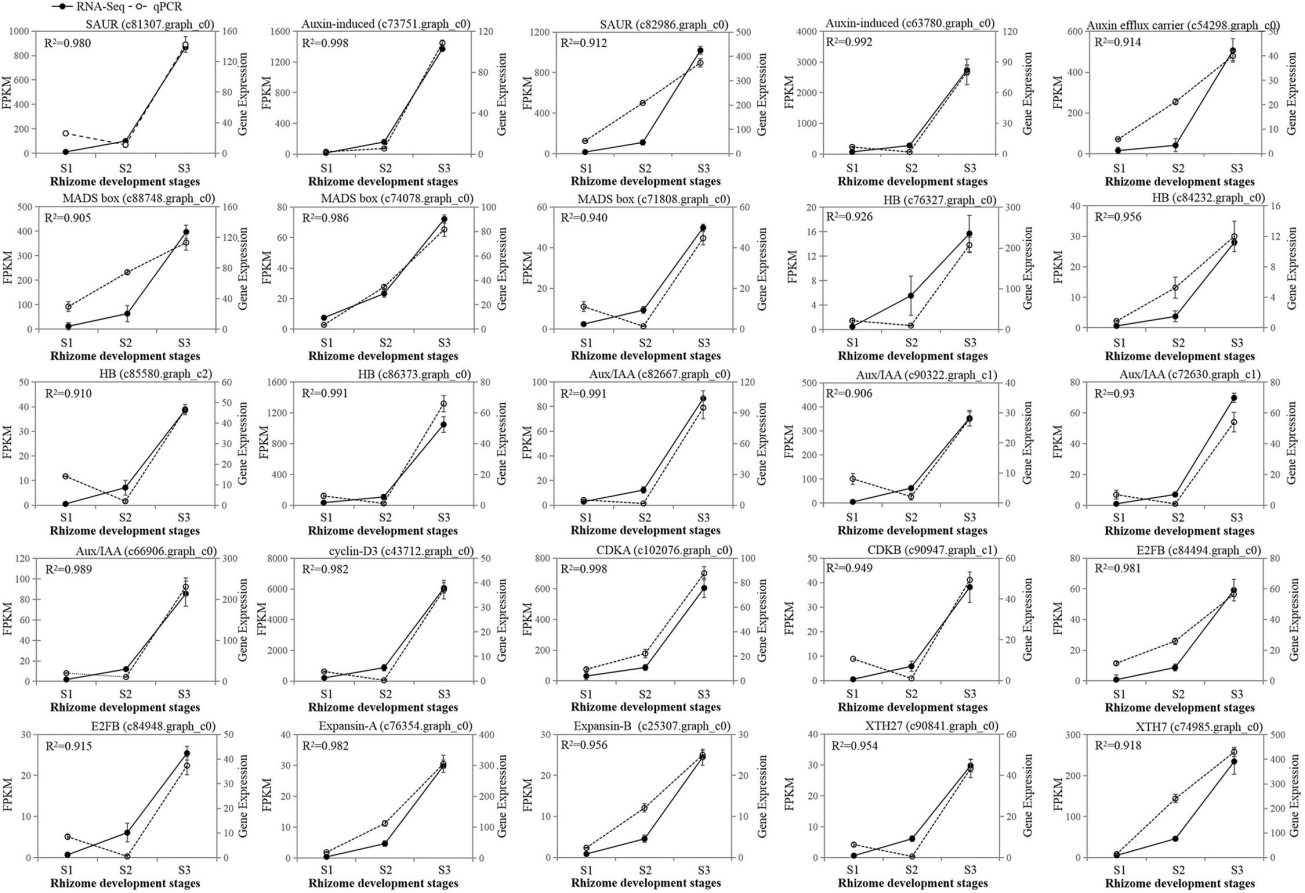
Validation of RNA-seq by qPCR. Stage 1 (S1), S2, and S3 represent the different enlargement stages of ginger rhizome development, respectively. The results represent the mean of three biological replicates with standard deviations. The solid black lines represent the RNA-seq results, and the dashed lines and white circles represent the qPCR results. *R*^2^ is the correlation coefficient between RNA-seq and qPCR.

qPCR was used to detect the expression levels of 25 randomly chosen potential regulatory DEGs (listed in bold type in S3–S5 Tables), including hormone-related genes, TFs, and cell division- and expansion-related genes that were upregulated during both the S1–S2 and S2–S3 transitions (Fig 7). The pairwise correlation coefficient was higher than 0.90, indicating that the qPCR results were in agreement with the RNA-seq data.

### Correlation analysis

To understand the regulatory network of rhizome enlargement, we conducted correlation tests between quantitative changes in hormone contents, RD, and transcripts in the three different developmental stages of *Zingiber officinale* ‘RY20’. The Pearson’s correlation analysis involved eight hormones, RD, and 264 transcripts (hormone biosynthesis and signalling pathway: 88; TFs: 120; and cell division and expansion: 56 transcripts; S3–S5 Tables).

The results of the correlation analysis between the hormone contents and RD showed that the levels of GA (*r* = 0.9971), ZT (*r* = 0.9946), IAA (*r* = 0.9794), and JA (*r* = 0.9577) were significantly positively correlated with RD (S8 Table). We found a significant correlation between two of the four hormones. Quantitative changes in IAA (*r* = 0.992), ZT (*r* = 0.9838), and JA (*r* = 0.9769) were significantly positively correlated with GA content. The quantitative changes in ZT (0.9533) and JA (0.9961) were significantly positively correlated with the IAA content, and the quantitative changes in JA (0.9228) were significantly positively correlated with the ZT content.

**Fig 8:**
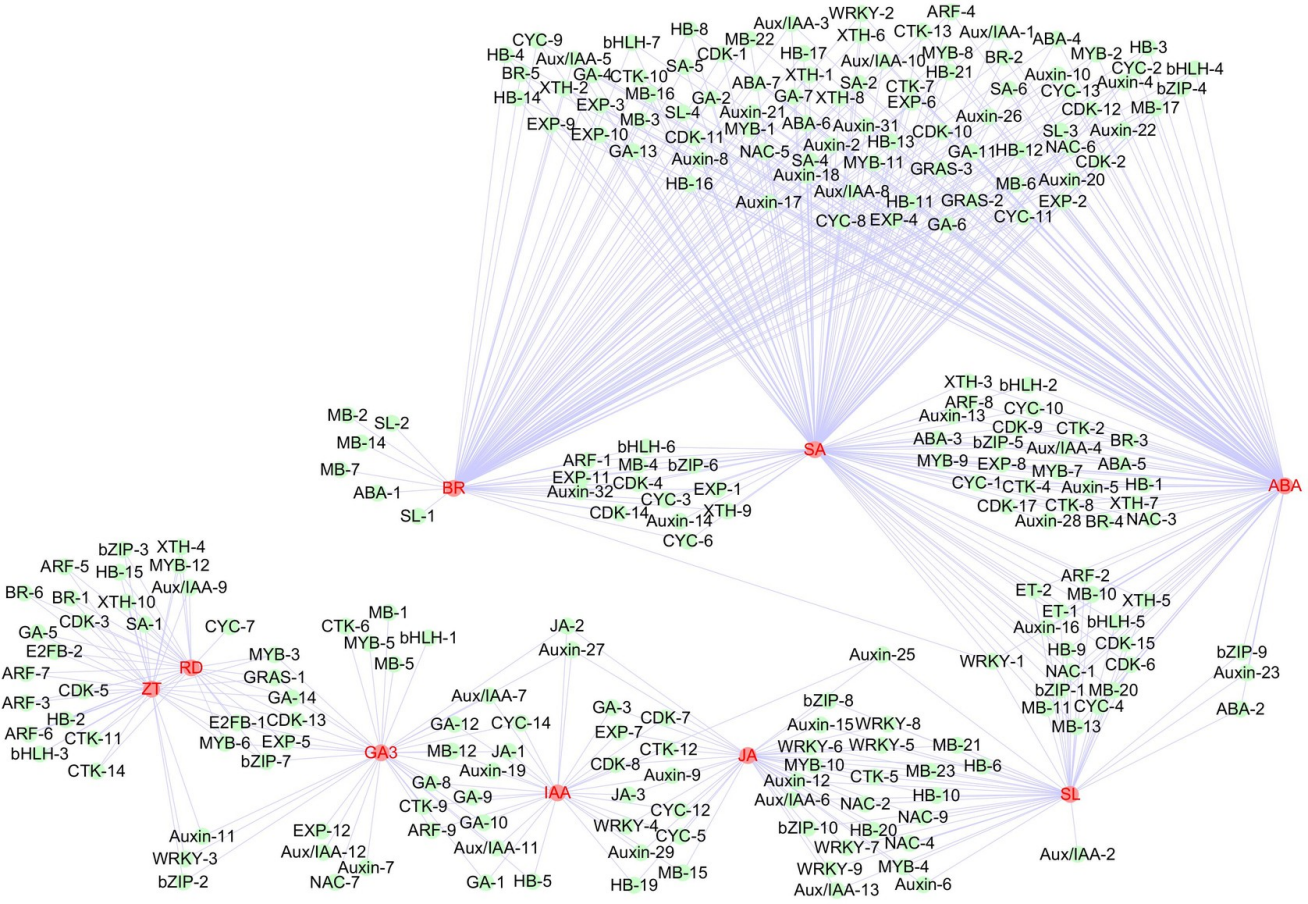
Connection network between hormones, rhizome diameter, and transcripts. The networks were visualised using the Cytoscape software (v. 3.7.2). The connection network between transcripts, including hormone synthesis and signalling-related genes, transcription factors, cell division and expansion-related genes, and hormones or rhizome diameter (RD), respectively. Symbol IDs are listed in S3–S5 Tables.

Correlation analysis between the changes in RD and the transcripts revealed that 30 genes, including seven hormone biosynthesis and signalling-related genes, 14 TFs, and nine cell division and expansion-related genes, were significantly positively correlated with RD (Fig 8 and S9 and S10 Tables). The seven genes implicated in hormone response, two cytokinin receptors, two GA-dioxygenases, a BR-6-oxidase, a BZR1, and a 6-methylsalicylic acid decarboxylase, were significantly upregulated through both the S1–S2 and S2–S3 transitions. Regarding TFs, a *GRAS*, two *HBs*, three *MYBs*, a *bHLH130*, two *bZIPs*, four *ARFs*, and an *IAA33* were also upregulated through both the S1–S2 and S2–S3 transitions. Of the nine genes implicated in cell division and expansion, a *CDK*, two *E2FBs*, an *EXP*, and two *XTHs* were upregulated through both the S1–S2 and S2–S3 transitions, and *CYC* and two *CDKs* were upregulated through the S1–S2 transition (Table 1).

**Table 1:** Differentially expressed genes (DEGs) correlated with rhizome diameter in *Zingiber officinale* Roscoe ‘RY20’.

Gene ID	FCS2/S1	FCS3/S2	Description	Symbol ID
c86223.graph_c1	3.24	2.45	cytokinin receptor	CTK-11
c74920.graph_c0	3.20	1.46	cytokinin receptor	CTK-14
c73572.graph_c0	2.44	1.67	gibberellin receptor GID1	GA-5
c70808.graph_c0	2.91	2.46	gibberellin 3-oxidase	GA-14
c82302.graph_c2	2.43	1.53	brassinosteroid-6-oxidase 2	BR-1
c82572.graph_c0	3.15	2.22	brassinosteroid resistant 1 BES1/BZR1 homolog protein 2	BR-6
c69542.graph_c0	2.24	1.07	6-methylsalicylic acid decarboxylase atA-like	SA-1
c48536.graph_c0	3.64	2.94	nodulation-signaling pathway 1 protein	GRAS-1
c76327.graph_c0	3.49	1.51	homeobox-leucine zipper protein HOX4-like	HB-2
c85580.graph_c2	3.64	2.47	homeobox protein knotted-1-like 3	HB-15
c39384.graph_c1	2.45	1.98	transcriptional activator Myb-like	MYB-3
c80203.graph_c0	2.65	2.29	transcription factor MYB122	MYB-6
c75645.graph_c0	3.53	2.74	transcription factor MYB108-like	MYB-12
c77122.graph_c0	2.68	1.53	transcription factor bHLH130	bHLH-3
c82361.graph_c0	2.49	1.94	bZIP transcription factor 53	bZIP-3
c80561.graph_c0	2.12	1.83	bZIP transcription factor 60	bZIP-7
c82676.graph_c0	2.28	1.78	auxin response factor 17-like	ARF-3
c74960.graph_c0	2.85	1.83	auxin response factor 2-like	ARF-5
c61359.graph_c0	3.28	2.17	auxin response factor 7	ARF-6
c89032.graph_c0	2.38	1.69	auxin response factor 17-like	ARF-7
c90322.graph_c1	3.42	2.36	auxin-responsive protein IAA33	Aux/IAA-9
c84481.graph_c0	2.11	0.69	mitotic-specific cyclin-2	CYC-7
c89925.graph_c0	1.84	0.95	Cyclin-dependent kinase C-2	CDK-3
c56621.graph_c0	2.45	0.88	Cyclin-dependent kinase B1-1	CDK-5
c90947.graph_c1	3.21	2.56	Cyclin-dependent kinase B2-1	CDK-13
c84494.graph_c0	3.32	2.64	transcription factor E2FB	E2FB-1
c84948.graph_c0	2.99	1.92	transcription factor E2FB-like	E2FB-2
c76354.graph_c0	3.34	2.73	Expansin-A1	EXP-5
c90841.graph_c0	3.34	2.19	XTH27	XTH-4
c74985.graph_c0	3.10	2.21	XTH7	XTH-10

Correlation analysis between the quantitative changes in hormone levels and the transcripts revealed that 36, 32, 30, 39, 130, 104, 141, and 45 genes were significantly (*r*^2^ > 0.9) correlated with GA_3_, ZT, IAA, JA, ABA, BR, SA, and SL, respectively (Fig 8 and S7 and S8 Tables). Among these genes, 36, 56, and 50 were significantly negatively correlated with ABA, BR, and SA, respectively, and the rest were positively correlated. A total of 94 genes had strong correlation coefficient values (*r*^2^ > 0.9) with GA_3_, ZT, IAA, and JA (S11 Table). Moreover, 33 out of 94 genes were correlated with more than two hormones at the same time. Of the 30 genes correlated with the RD, 19 were commonly correlated with ZT, such as CRE1, CRE2, HB, two ARFs, CDKB1, E2FB2, and XTH7, etc., as well as eight genes (GA3ox, GRAS, MYB, MYB122, bZIP60, CDKB2, E2FB1, and EXPA1) were commonly correlated with GA and ZT (S11 Table).

### Comparison of gene expression levels in different RD ginger varieties

The RD of 57 ginger varieties were investigated and ranged from 21.68 mm to 39.93 mm (S1 Table). 15 typical varieties (5 large (37.39 ≤ RD ≤ 39.93 mm), 5 medium (29.94 ≤ RD ≤ 31.70 mm) and 5 small (21.68 ≤ RD ≤ 25.04 mm)) were selected for further study (listed in bold type in S1 Table and S1 Fig).

**Fig 9:**
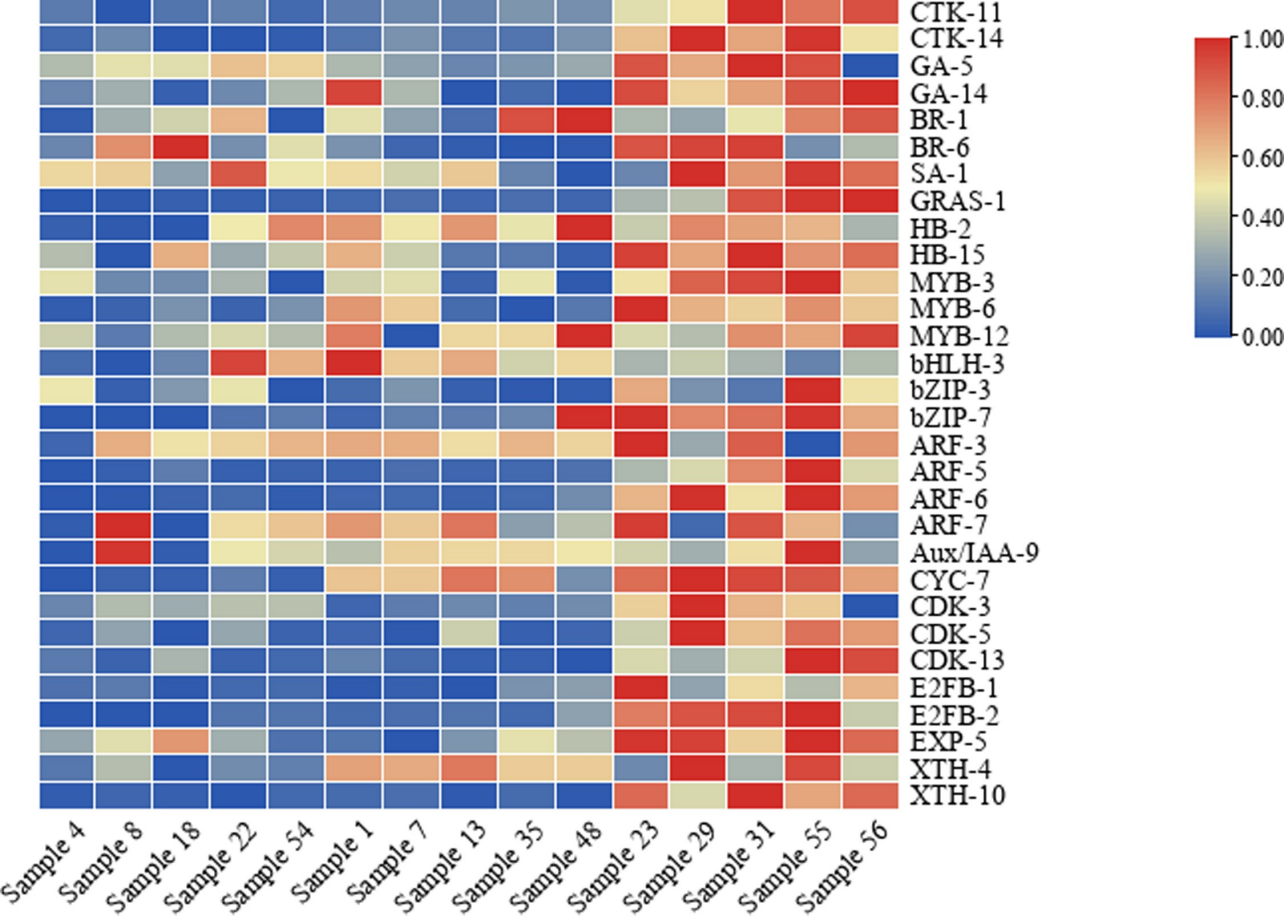
Expression profifiling of 30 related genes in 15 different ginger varieties. Expression analysis of 30 genes at peak enlargement stage S3 (almost grow to mature size) of ginger rhizomes were determined by qPCR and illustrated by heatmap. The Min-Max method was used to standardize the relative expression data of each gene, and the transformation formula: (n-minimum value)/(maximum value-minimum value) was used to make all trait values in a specific interval of 0–1, where n is independent variables.

The expression levels of 30 correlated genes were valued and compared in 15 different ginger varieties. The majority of 30 genes displayed the highest transcript accumulation in rhizomes of large RD varieties (Simple 23, 29, 31, 55 and 56), and obvious lower in mRNA levels in medium (Simple 1, 7, 13, 35 and 48) and small RD varieties (Simple 4, 8, 18, 22 and 54) (Fig 9). Some genes showed distinct RD-specific expression patterns across the 15 different ginger varieties examined (Fig 9), indicating their candidate roles in the regulation of rhizome growth.

Correlation analysis of the RD values of 15 different ginger varieties and the expression of 30 correlated genes revealed that the expression levels of the following 17 genes were significantly (*r* >0.6) positively correlated with RD (S12 Table): two cytokinin receptors *CRE1/CRE2* (0.8124 and 0.8506), *GA3ox* (0.7245), *GRAS* (0.7541), *homeobox protein knotted-1* (*HB*; 0.6573), *MYB* and *MYB122* (0.6929 and 0.7912), *bZIP60* (0.7950), two ARFs (0.7283 and 0.8441), *CYC2* (0.8883), *CDKB1* and *CDKB2* (0.7307 and 0.6569), *E2FB1* and *E2FB2* (0.7538 and 0.8285), *EXPA1* (0.6479), and *XTH7* (0.8381).

Among these genes, interestingly, the expression levels of *CRE1* (0.6113), *HB* (0.7793), and *EXPA1* (0.6930) were significantly positively correlated with the expression of *CRE2*; the expression levels of *HB* (0.6989), *MYB* (0.7183), and *CDKB1* (0.7892) were significantly (*r* >0.6) positively correlated with the expression of *bZIP60*; the expression levels of *CDKB1* (0.6204), *CDKB2* (0.7126), *E2FB1* (0.6392), and *E2FB2* (0.7812) were significantly (*r* >0.6) positively correlated with the expression of *CYC2*; the expression levels of *MYB* (0.6084) and *CDKB1* (0.6283) were significantly (*r* >0.6) positively correlated with the expression of *CDKB2*; and the expression levels of *CDKB1* (0.7272) and *E2FB1* (0.6134) were significantly (*r* >0.6) positively correlated with the expression of *E2FB2* (S12 Table).

## Discussion

### Roles of GA and cytokinin during rhizome enlargement

Cytokinins and GAs are the two main hormones that promote plant growth by regulating cell division and cell growth [32]. In this study, the transcriptional dynamics at three distinct developmental stages of ginger rhizomes were monitored. The ‘Zeatin biosynthesis’ GO term was significantly upregulated during the S1–S2 transition (Fig 4). The expression of cytokinin receptors was upregulated through both the S1–S2 and S2–S3 transitions, whereas an increase in ZT was detected during S1–S3 (Fig 1C and S5 Table). These data suggest that cytokinin may play a positive role in rhizome enlargement, which is consistent with the induction of tuber development by cytokinin in potato [33]. Studies on the influence of GA on tuber formation have revealed that GA can inhibit the formation of tubers [7, 34]. Transgenic potato plants with the reduced expression of *StGA2ox1*, a GA-degradation gene, can increase GA20 levels and exhibit altered stolon expansion phenotypes and delayed tuber formation [35]. However, other studies have found that endogenous GAs are closely related to tuber enlargement in Chinese yam, and GA treatment can increase tuber yield [36]. In our study, an increase in GA_3_ was detected during S1–S3, as well as an increase in cytokinin, which was consistent with the upregulation of the GA biosynthesis genes *GA3ox* and *GA20oxs*, as well as GA-signalling, GA-receptor, GA-responsive, and GA-regulated genes (Fig 1C and S5 Table). Interestingly, *GA2ox* expression was upregulated, whereas GA content gradually increased. This phenomenon may be due to the synthesis of GA by upregulated biosynthesis genes more than the degradation by *GA2oxs*. According to previous reports, *GA13ox* increases or reduces GA activity by catalysing the conversion of GA9 to GA20 [37] or the conversion of GA4 to GA1, respectively, and participating in GA homeostasis [38]. Our transcriptome data show that *GA13ox* was upregulated through the S1–S2 transition and downregulated through the S2–S3 transition, along with some GA-receptor, GA-responsive, and GA-regulated genes (S5 Table). Thus, we speculated that *GA13ox* may play a role in fine-tuning rhizome enlargement by decreasing GA bioactivity in the late stage of development.

The interaction between GA and cytokinin occurs during trichome initiation, the resumption of cellular activity, and other plant growth and development processes [39, 40]. Furthermore, the interaction between cytokinin and GA has also been reported in the reactivation of meristem activity in potato tubers [41]. Interestingly, the contents of GA (*r* = 0.9971) and ZT (*r* = 0.9946) were significantly positively correlated with the RD, and the quantitative change in ZT (*r* = 0.9838) was significantly positively correlated with GA (S8 Table). In our transcriptome data, two cytokinin-N- glucosyltransferase genes in the cytokinin pathway, all induced during S1–S3, were significantly correlated with GA (S5 Table). DEGs in the GA pathway that were induced during S1–S3, including *GA3ox* and *gibberellin receptor GID1*, were significantly correlated with cytokinin (S5 Table). Among them, *GA3ox* was significantly positively correlated with RD. Therefore, we postulate that GA might play common and vital positive roles alongside cytokinin in regulating developmental processes that lead to rhizome enlargement.

### Other hormone changes in ginger rhizome enlargement

As a basic regulatory factor, auxin participates in the process of modified root or stem development [6, 42]. Wu et al. [43] found that auxin regulates the growth and development of carrots in a tissue-specific and stage-dependent manner through changes in the IAA levels during development. SAUR proteins were found to be mainly involved in regulating the synthesis and transport of auxin, thereby affecting the expansion of cells [44]. ARF is a TF that recognises and binds to auxin response elements and regulates the expression of auxin response genes [45]. According to our transcriptome data, the ‘Auxin signalling pathway’ GO term was significantly upregulated through the S2–S3 transition (Fig 4). *SAURs* and other auxin synthesis- or signalling-related genes were expressed in both upregulation and downregulation during S1–S2, whereas most of these genes were upregulated during S2–S3 (S5 Table). The difference in expression between the two transitions may be due to the different means of enlargement, and auxin may play a more important role in the S2–S3 transition than in the S1–S2 transition, which is consistent with the greater increase in the content of the endogenous hormone IAA. Correlation analysis revealed that the expression of ARFs (c74960.graph_c0 and c61359.graph_c0), which were upregulated during S1–S3, was significantly positively correlated with the RD (S11 Table). This indicates that SAURs may affect cell expansion through the synthesis and transport of auxin under the regulation of ARFs, which are conserved in the plant kingdom [43, 45], and function in the regulation of rhizome enlargement in ginger. Liu et al. found that the expression of genes related to auxin signaling pathway was higher in young ginger tissues and lower in mature and old tissues [46]. The reason for the inconsistent results may be that the rhizome enlargement period we studied was before the maturity period of Liu et al. No more auxin is required after the completion of the expansion process, so auxin-related gene expression is reduced in mature and old tissues. Together, our and Liu et al.’s results showed that auxin and its related genes play an important role in the early cell expansion and plant growth of ginger rhizomes.

As positive regulators of potato tuber formation, ABA and JA accumulate in leaves and stolons/tubes during the entire tuber development process [47, 48]. SLs could also have an effect—either alone or in combination with other phytohormones—in controlling stolon development and maintaining tuber dormancy [11]. BR, ET, and SA usually play roles in the adaptation response to environmental stressors and the regulation of plant senescence [49–51], and their roles in modified root or stem development are still unclear. However, BRs have been shown to play an important role in controlling cell elongation and can induce the expression of SAUR genes [52, 53]. In Liu et al’s study, most DEGs enriched in plant hormone signals such as ethylene, BRs, JA and SAwere relatively highly expressed in young ginger rhizomes, and played important roles in ginger rhizome growth [46]. In our study, the JA content increased with the development of ginger, which is consistent with the upregulation of all three JA biosynthesis-related JA O-methyltransferase and JAR genes through the S1–S2 and S2–S3 transitions (Fig 1C and S5 Table). In contrast, the hormones ABA, SA, BR, and SL first decreased and then increased, and more DEGs related to these hormones, including an ABA-responsive element-binding factor, two ABA-activated protein kinases, an ABA 8’-hydroxylase, two ABIs, three BRI1s, two UDP-glycosyltransferases, and two SL esterases, were indeed upregulated through the S2–S3 transition (Fig 1C and S5 Table). ET-responsive TF ERF and ET-insensitive protein genes were also induced through the S2–S3 transition (S5 Table). Furthermore, correlation analysis revealed that the expression of *BR-6-oxidase* (c82302.graph_c2), *BR-resistant 1 BES1/BZR1* (c82572.graph_c0), and 6-methylsalicylic acid decarboxylase (c69542.graph_c0), which were upregulated during S1–S3, were significantly positively correlated with RD (S11 Table). Thus, we speculated that they may play a complicated regulatory role in ginger rhizome enlargement, as well as serve as an important focus for future studies.

### Transcription factors and cell division or expansion-related genes regulate rhizome enlargement in ginger

MADS-box and HB family genes were mainly upregulated during the enlargement of the ginger rhizome in our study, which may enhance the expression of rhizome formation-related genes, and this finding is consistent with previous studies [15]. Notably, the expression of most auxin-related TFs, such as Aux/IAA and ARF family genes, was also upregulated through both the S1–S2 and S2–S3 transitions (Fig 6 and S6 Table). A large number of StIAA genes were found to be highly expressed in the stolon organs and during the tuber initiation and developmental stages, and most of these genes were responsive to IAA treatment [54]. Thus, we postulate that Aux/IAA TFs may also play an important role in regulating the rhizome enlargement of ginger in response to high IAA levels. The common TFs MYB, WRKY, NAC, and bZIP are involved in many biological processes, including plant growth and development, primary and secondary metabolic reactions, cell morphology and model formation, responses to biotic and abiotic stresses, and hormone signal transduction [55]. However, at present, the roles of MYB, WRKY, NAC, bHLH, bZIP, and GRAS in modified root or stem development remain poorly defined [55–57]. In our study, *MYB*, *WRKY*, *NAC*, *bHLH*, *bZIP*, and *GRAS* family genes were also induced in the ginger rhizome enlargement process (Fig 6 and S6 Table). The expression patterns of some TFs in S1–S2 and S2–S3 were opposite, which may indicate that the TFs perform different functions during the two transition periods. It would be interesting to investigate how TFs regulate rhizome enlargement in ginger, although they are known to have many other functions.

Interestingly, the expression levels of genes involved in cell division or expansion, such as *CDKs*, *CYCs*, *E2FBs*, *EXPs*, and *XTHs*, were also significantly altered in our study (S7 Table). Most of the *CycDs* were upregulated through the S2–S3 transition, and both *CDKBs* and *E2FB* were upregulated during S1–S3 (S7 Table). These results suggest that *CycDs*, *CDKBs*, and *E2FB* may play positive roles in rhizome enlargement by regulating cell division, which is in accordance with previous findings that tuber or rhizome enlargement was improved in potato by *StCDKB* [58], in radish by *CycD3* [20], and in tuber mustard by E2Fs (*E2Fa*, *E2Fb*, and *E2Fc*) [21]. *RsEXPB1* [59] and *BjXTH1* [21] promoted tuber formation in radish and tuber mustard, respectively. However, according to our transcriptome data, most of the *EXPs* and *XTHs* were expressed in both upregulation and downregulation through the S1–S2 and S2–S3 transitions, while two *EXPs* and two *XTHs* were upregulated during S1–S3 (S7 Table). Therefore, we speculate that different *EXPs* and *XTHs* may play roles in different stages of ginger rhizome enlargement.

### Possible regulatory network in ginger rhizome enlargement process

Correlation analysis between the hormone contents and RD showed that the contents of GA, ZT, IAA, and JA were significantly positively correlated with the RD (S8 Table), which suggests that rhizome enlargement may be mainly dependent on the control of GA, cytokinin, auxin, and JA biosynthesis. Correlation analysis between the changes in the RD and transcripts revealed that 30 genes were significantly correlated with RD (Fig 8 and S9 and S10 Tables). Further correlation analysis between the 30 correlated genes and the RD of 15 different ginger varieties showed that the expression levels of *CRE1*, *CRE2*, *GA3ox*, *GRAS*, *HB*, *MYB*, *MYB122*, *bZIP60*, two ARFs, *CYC2*, *CDKB1*, *CDKB2*, *E2FB1*, *E2FB2*, *EXPA1*, and *XTH7* were significantly positively correlated with RD (S10 Table), suggesting that these may be key genes regulating rhizome enlargement. However, simple correlation analysis cannot accurately determine the true relationship between genes and RD or yield; further confirmation is required.

**Fig 10:**
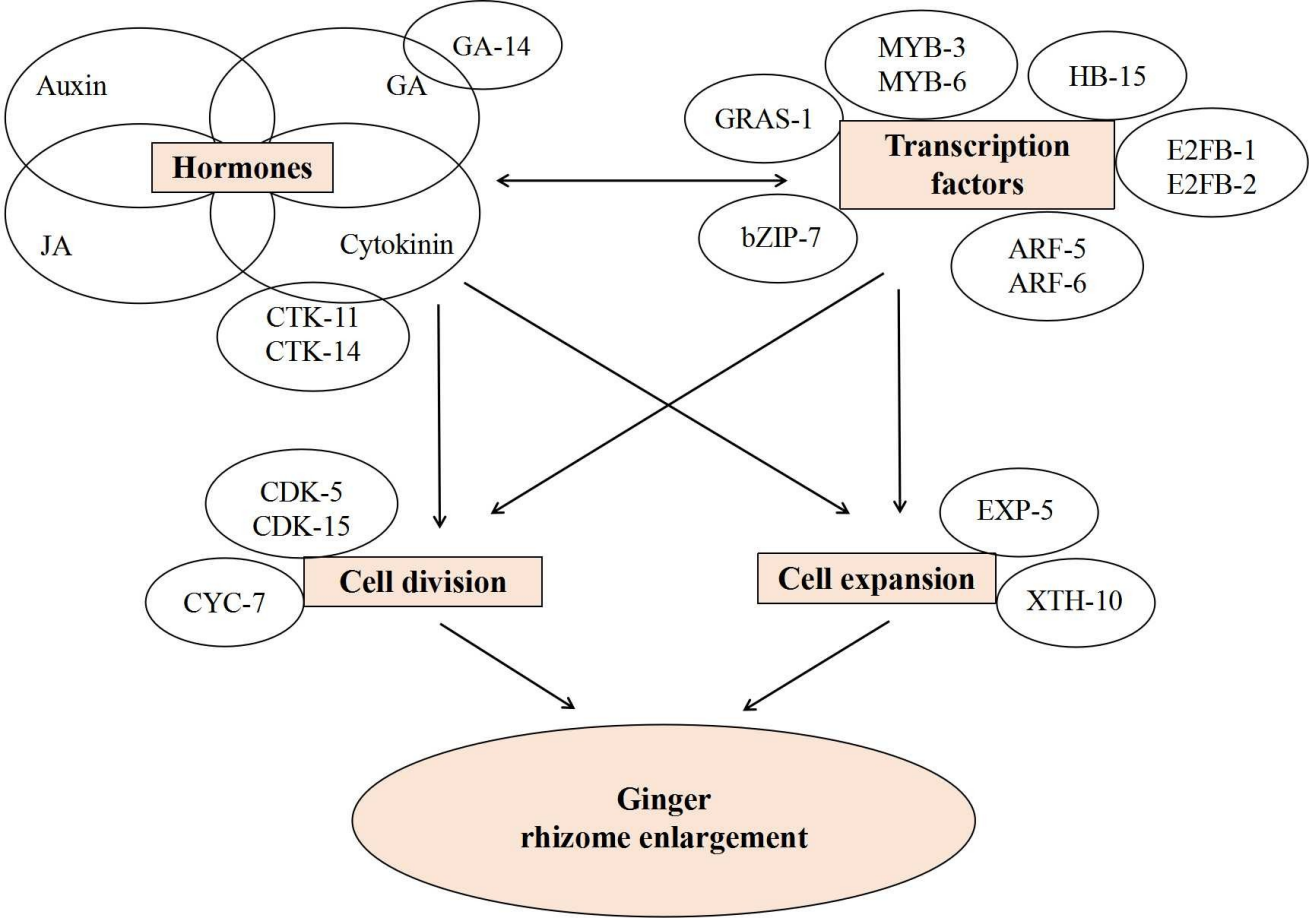
Possible positive regulatory network in ginger rhizome enlargement process.

Genes related to cell division or expansion are usually regulated by hormones and TFs. For example, the expression of *CYC* is regulated by plant growth factors, such as cytokinin, auxin, BR, and GA [18], and CYCs are induced by the E2F transcription factor and then bind to CDKs, interacting with them to participate in cell division [60]. TFs can regulate the synthesis and transduction of hormones, while being regulated by plant hormones [2]. In our study, correlation analysis revealed that TFs and the genes related to cell division or expansion had a strong correlation coefficient values with GA_3_, ZT, IAA, and JA, and the genes related to cell division or expansion were significantly correlated with TFs (S11 and S12 Tables). Of the 17 key genes, the TFs (*HB*, *bZIP60*, *E2FB1*, *E2FB2*, *GRAS*, *MYB*, *MYB122*, and two ARFs) and genes related to cell division and expansion (*CDKB1*, *CDKB2*, *EXPA1*, and *XTH7*) were commonly correlated with ZT and GA (S11 Table). At the same time, the genes related to cell division or expansion (*CYC2*, *CDKB1*, *CDKB2*, *EXPA1*, and *XTH7*) were significantly correlated with TFs (*HB*, *MYB*, *bZIP60*, *E2FB1*, and *E2FB2*) (S12 Table). Therefore, we speculate that the possible positive regulatory network in the ginger rhizome enlargement process is as follows (Fig 10): the *HB*, *MYB*, *bZIP60*, *E2FB1*, and *E2FB2* TFs may regulate the synthesis and transduction of the hormones GA_3_, ZT, IAA, and JA, while being regulated by them. They may be involved in cell division or expansion by individually or jointly regulating the *CYC2*, *CDKB1*, *CDKB2*, *EXPA1*, and *XTH7* genes and ultimately promote the enlargement of ginger rhizomes. The relationships among these TFs, genes related to cell division or expansion, and hormones need to be clarified in the future.

## Conclusions

The results of our study show that the content of endogenous hormones, mainly cytokinins, GA, auxin, and JA, were enhanced during rhizome enlargement. Transcriptome data and correlation analysis suggested that ginger rhizome enlargement may be mainly related to the regulation of endogenous cytokinin, GA_3_, auxin, and JA biosynthesis pathways and signal transduction; *GRAS*, *HB*, *MYB*, *MYB122*, *bZIP60*, *ARF1*, *ARF2*, *E2FB1*, and *E2FB2*, which may regulate the expression of rhizome formation-related genes; and *CYC2*, *CDKB1*, *CDKB2*, *EXPA1*, and *XTH7*, which may mediate cell division and expansion. Therefore, our results provide new insights into rhizome or tuber enlargement in horticultural crops and provide useful gene resources for future development, through genetic engineering, of new ginger varieties with increased RD and yield.

## Supporting information

S1 Fig Bulbs of 15 different ginger varieties. The ginger bulbs of the first main branch of 15 different ginger varieties at peak enlargement stage S3 (almost grow to mature size). Bar = 35 mm.
(TIF)

S1 Table Diameters and weights of bulbs of different ginger varieties. (DOCX)

S2 Table Retention times, character ions and collision energies for protonated or deprotonated plant hormones ([M+H]+or[M−H]-). (DOCX)

S3 Table Primer sequences used for qPCR of each DEG. (DOCX)

S4 Table RNA-seq data statistics of annotation results for ginger unigenes. (DOCX)

S5 Table Unigenes associated with the hormone biosynthesis and signalling pathway exhibiting a |log2 FC ≥ 1| and *p* ≤ 0.05 in at least one transition. (DOCX)

S6 Table Unigenes associated with transcription factors exhibiting a |log2 FC ≥ 1| and *p* ≤ 0.05 in at least one transition. (DOCX)

S7 Table Unigenes associated with cell division and expansion exhibiting a |log2 FC ≥ 1| and *p* ≤ 0.05 in at least one transition. (DOCX)

S8 Table Correlation analysis between the hormone contents and rhizome diameter. (DOCX)

S9 Table Correlation matrix of hormone contents/rhizome diameter and gene expression levels. (XLSX)

S10 Table Interaction value between hormone contents, rhizome diameter, and transcripts. (XLSX)

S11 Table DEGs correlated with GA, ZT, IAA, JA, and rhizome diameter. (XLSX)

S12 Table Correlation analysis between the rhizome diameter of different ginger varieties and the expression of 30 DEGs. (XLSX)
