# Supplementary figures and images for: Dynamic transcriptome profiling provides insights into rhizome enlargement in ginger (Zingiber officinale Rosc.)
Source: PLoS One. 2023 Jul 14;18(7):e0287969. doi: 10.1371/journal.pone.0287969 (PMC10348538; doi:10.1371/journal.pone.0287969)

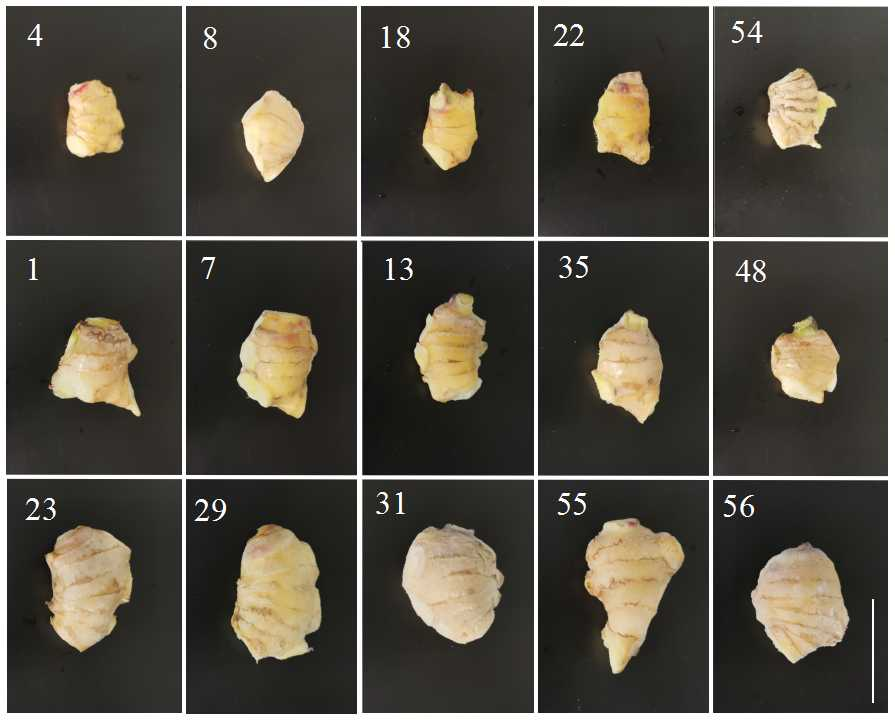

Supplement: S1 Fig — The ginger bulbs of the first main branch of 15 different ginger varieties at peak enlargement stage S3 (almost grow to mature size). Bar = 35 mm. (TIF) [file pone.0287969.s001.tif]
