# Supplementary material for: Dynamic transcriptome profiling provides insights into rhizome enlargement in ginger (Zingiber officinale Rosc.)
Source: PLoS One. 2023 Jul 14;18(7):e0287969. doi: 10.1371/journal.pone.0287969 (PMC10348538; doi:10.1371/journal.pone.0287969)
Supplement: S1 Table — (DOCX) [file pone.0287969.s002.docx]

**S1 Table. Diameter and weight of ginger bulbs of different ginger varieties**

| **No.** | **Variety** | **Diameter (mm)** | **Weight (g)** | **No.** | **Variety** | **Diameter (mm)** | **Weight (g)** |
| --- | --- | --- | --- | --- | --- | --- | --- |
| **1** | **Leshan White Ginger** | **31.27±1.62** | **21.54±0.58** | 30 | Guangxi Ginger | 36.16±3.90 | 26.77±1.06 |
| 2 | Guangxi Ginger | 36.59±5.61 | 35.17±1.58 | **31** | **Thai White Ginger** | **37.96±0.77** | **35.58±2.07** |
| 3 | Tongling White Ginger 1 | 28.82±2.25 | 22.56±1.92 | 32 | Guangxi Guilin Vine Ginger | 26.61±2.21 | 16.60±1.10 |
| **4** | **Zengcheng Yellow Ginger** | **22.67±0.85** | **9.92±0.30** | 33 | Ry 57 | 26.76±0.76 | 16.77±1.46 |
| 5 | Enshi Crested Ginger | 28.07±2.07 | 20.34±1.61 | 34 | Wujian Yellow Ginger | 27.52±1.63 | 17.47±1.56 |
| 6 | Shandong Small Ginger | 28.91±1.66 | 19.92±2.15 | **35** | **Shandong Ginger** | **31.70±0.73** | **31.26±2.87** |
| **7** | **Su Xianshi Small Yellow Ginger** | **30.41±1.44** | **23.35±1.66** | 36 | Guizhou Yellow Ginger | 27.58±1.72 | 17.69±1.76 |
| **8** | **Jiangxi Small Yellow Ginger** | **24.58±2.87** | **21.22±1.35** | 37 | Zhejiang Small Ginger 1 | 25.45±3.24 | 12.89±1.61 |
| 9 | Fraternity Ginger | 28.19±3.42 | 18.30±1.44 | 38 | Zhugen Ginger | 28.30±4.41 | 16.14±1.62 |
| 10 | Anhui Huangshan Ginger | 26.70±0.79 | 21.46±3.55 | 39 | Jiangxi Small Yellow Ginger | 27.11±1.37 | 20.16±1.15 |
| 11 | Leshan Yellow Ginger 1 | 25.39±6.61 | 19.71±2.58 | 40 | Guangdong Meizhou White Ginger | 25.76±2.81 | 12.13±1.66 |
| 12 | Tongling Yellow Ginger | 26.62±2.21 | 15.94±1.18 | 41 | Hunan Ginger | 25.36±2.47 | 11.39±0.97 |
| **13** | **Hubei Xuan'En Ginger 1** | **29.94±1.66** | **19.05±1.30** | 42 | Yunnan Pingbian Yellow Ginger | 28.69±3.66 | 22.50±2.52 |
| 14 | Hunan Longhui Ginger 1 | 26.25±2.69 | 14.84±1.76 | 43 | Jiangxi Small Ginger | 25.04±0.33 | 17.28±1.64 |
| 15 | Hubei Xuan'En Ginger 2 | 26.29±1.30 | 15.30±0.50 | 44 | Huangguoshu Ginger | 26.37±1.44 | 14.30±2.23 |
| 16 | Chaling Yellow Ginger | 26.42±4.83 | 13.16±1.17 | 45 | Zhejiang Pan'An Ginger | 29.07±5.58 | 22.78±6.45 |
| 17 | Guangxi Fragrant Ginger | 25.16±3.09 | 13.24±2.57 | 46 | Hunan Longhui Ginger 2 | 26.62±5.18 | 15.49±0.93 |
| **18** | **Sichuan Chengdu Erhuang Ginger** | **23.80±1.21** | **11.76±1.16** | 47 | Tongling White Ginger 2 | 26.64±0.72 | 15.08±1.29 |
| 19 | Ry 22 | 26.32±2.43 | 14.09±1.37 | **48** | **Jiangxi Yichun Ginger** | **30.12±0.65** | **17.22±1.25** |
| 20 | Wuzhiyan Yellow Ginger | 29.42±2.92 | 20.07±0.28 | 49 | Leshan Small White Ginger | 25.84±1.56 | 14.13±1.17 |
| 21 | Taiwan 1 | 28.34±2.65 | 22.74±1.91 | 50 | Zhejiang Ruian Ginger | 27.56±1.95 | 15.09±0.90 |
| **22** | **Hebei Small Ginger 2** | **21.68±0.12** | **8.78±0.56** | 51 | Yujiang 1 | 25.94±2.27 | 16.30±1.05 |
| **23** | **Sichuan Ginger** | **39.93±0.76** | **44.46±1.99** | 52 | Yunnan Wenshan Small Yellow Ginger 2 | 27.57±5.04 | 14.65±1.76 |
| 24 | Luoping Small Yellow Ginger | 25.88±0.13 | 15.78±2.10 | 53 | Fujian Ginger | 26.06±0.04 | 9.79±1.07 |
| 25 | Leshan Yellow Ginger 2 | 28.16±2.45 | 13.86±1.61 | **54** | **Hunan Longhui Ginger 3** | **25.01±1.15** | **13.73±2.04** |
| 26 | Sichuan Zhugen Ginger | 25.48±3.32 | 17.42±2.00 | **55** | **Anqiu Ginger** | **37.39±4.45** | **53.89±1.99** |
| 27 | Ry 20 | 33.12±1.88 | 25.76±1.19 | **56** | **Anqiu Yellow Ginger** | **39.52±1.82** | **45.41±2.30** |
| 28 | Zhejiang Yongkang Yellow Ginger | 26.43±1.45 | 16.89±2.27 | 57 | Hubei Yellow Ginger | 28.77±0.62 | 24.47±1.91 |
| **29** | **Burmese Ginger** | **39.68±0.17** | **58.47±4.90** |  |  |  |  |
