# Supplementary material for: Dynamic transcriptome profiling provides insights into rhizome enlargement in ginger (Zingiber officinale Rosc.)
Source: PLoS One. 2023 Jul 14;18(7):e0287969. doi: 10.1371/journal.pone.0287969 (PMC10348538; doi:10.1371/journal.pone.0287969)
Supplement: S2 Table — (DOCX) [file pone.0287969.s003.docx]

**S2** **Table. Retention times, character ions and collision energies for protonated or deprotonated plant hormones ([M+H]+or[M−H]-)**

| **Plant hormones** | **Polarity** | **Mother ion (m/z)** | **Daughter ion (m/z)** | **Quantification ion** | **Collision energy (eV)** | **Retention time (min)** |
| --- | --- | --- | --- | --- | --- | --- |
| GA | **-** | 345.1 | 143.1/239.2 | 345.1/143.1 | 25/13 | 2.6 |
| IAA | **+** | 176.1 | 130.1/76.9 | 176.1/130.1 | 13/40 | 3.1 |
| JA | **-** | 209.1 | 58.9 | 209.1/58.9 | 5 | 5.6 |
| SA | **-** | 137.0 | 93/65 | 137.0/93 | 13/25 | 3.8 |
| ABA | **-** | 263.1 | 219/201 | 263.1/219 | 9/13 | 4.3 |
| ZT | **-** | 352.3 | 220.2/202.1 | 352.3/220.2 | 23/17 | 5.0 |
| BRs | **+** | 481.3 | 445.2/94.9 | 481.3/445.2 | 9/37 | 9.1 |
| SLs | **+** | 347.1 | 215.1/97.1 | 347.1/215.1 | 23/15 | 4.5 |
