# Supplementary material for: Dynamic transcriptome profiling provides insights into rhizome enlargement in ginger (Zingiber officinale Rosc.)
Source: PLoS One. 2023 Jul 14;18(7):e0287969. doi: 10.1371/journal.pone.0287969 (PMC10348538; doi:10.1371/journal.pone.0287969)
Supplement: S3 Table — (DOCX) [file pone.0287969.s004.docx]

**S3** **Table. Primer sequences used for qPCR of each DEGs**

| **Genes** | **Primer sequences (5’- 3’)** | **Genes** | **Primer sequences (5’- 3’)** |
| --- | --- | --- | --- |
| *Actin* | Forward: AGATGCCCAGAGGTCCTTTT  Reverse: CATAGTTGATCCACCGCTGA | c82667.graph_c0 | Forward: ATACCAGTGTTGGTGAAGGA  Reverse: TGAAGGTGAGGATGGACAGT |
| c81307.graph_c0 | Forward: TTTGCGGATCGCCGACATCGAT  Reverse: TTCATGTTGTGGATGCTTTCCA | c90322.graph_c1 | Forward: AGCACAGATCAAGCCAGTT  Reverse: AGCCTGAACATGTTTCTCTGA |
| c73751.graph_c0 | Forward: TTCTCTTCCTTGTCTCGTCT  Reverse: TGGTGGAGAGTCCTTTGCGT | c72630.graph_c1 | Forward: TGTCTGGAAGCTGGTGTTTGA  Reverse: TCCAGTTGCAGAGCAAAGGA |
| c82986.graph_c0 | Forward: TTGCCGTCGCAGGTGTACACT  Reverse: ACGAACTCATTGATTCCGGA | c66906.graph_c0 | Forward: AAGGAGCTGTCACTAGCACT  Reverse: TCTTTGTCCTCGTAGGTAAGA |
| c63780.graph_c0 | Forward: ATTCTGATGGGAGAGAAGT  Reverse: ACCGGACTCCTCGTAGCGAA | c43712.graph_c0 | Forward: ATCCATGGCTTGTCGTCCT  Reverse: TTACCTCCAATCGGCGAGGAA |
| c54298.graph_c0 | Forward: TGAACGGATGAAAAGGAAGA  Reverse: ACTTCGCCTTTAAGAGGAT | c102076.graph_c0 | Forward: TCTCGAATAGCAGTGCTAGGA  Reverse: AGGGTACTTATGGTATTGTGT |
| c88748.graph_c0 | Forward: ATAGACTTCCGGTTGCCTCT  Reverse: AGTAATCGAGAGTAGAGGAAGA | c90947.graph_c1 | Forward: AGACATTTGGTCGCTTGGAT  Reverse: TCGCAAGCATTCCTTGATCA |
| c74078.graph_c0 | Forward: TGGTGCACAGGCTGATTGCA  Reverse: TGTTCAGTCATGGCCAGCAACT | c84494.graph_c0 | Forward: AGAAGTTCCTGATCCTGATGA  Reverse: ACTCATCAAACTTCTCTTCA |
| c71808.graph_c0 | Forward: AGAAGAACCATTCACATCCA  Reverse: ACAGACACAAGCTTACTACT | c84948.graph_c0 | Forward: AGAAAGGATGAGGGTGTTCA  Reverse: AGGGTAGTACCATGAGGTGCT |
| c76327.graph_c0 | Forward: TGGAATTAACATCTTCGGTGA  Reverse: ATGTGATCTTGACTTGAACA | c76354.graph_c0 | Forward: TAGTGATCTCGGTGGTGGCT  Reverse: TGCGCGAACAGATCACTATA |
| c84232.graph_c0 | Forward: ACATGTGCTATCCTATGCAA  Reverse: AGTATTTGGTGATGGAATCA | c25307.graph_c0 | Forward: ATCGCAACGGCTACCAGTCT  Reverse: AATTCGCACCCTGGCGAAAT |
| c85580.graph_c2 | Forward: TCATCACTGAGAGAACTAAGCT  Reverse: TCTGGGTTGCACCCATGAGA | c90841.graph_c0 | Forward: TGTTGGTCTTCGGCAAAGTGT  Reverse: TCGCGGAGCAAGATGAGGTT |
| c86373.graph_c0 | Forward: TGGAGAACATCAAAGCCATCGT  Reverse: TTGCCTGTTTCGCTCAGCA | c74985.graph_c0 | Forward: AACCGACGATACACTCTT  Reverse: ACCTTCATACCGGCAATTGA |
| c86223.graph_c1 | Forward: AAGGAGGAACTTAACCGAGT  Reverse: ACCTTGGAATCTTTTGTGCCT | c39384.graph_c1 | Forward: TCGTCAGGGACATAGCCATGT  Reverse: AAGACAGGGATCTTCTGCGT |
| c74920.graph_c0 | Forward: TGACGACGCCGTAATAGAGCTT  Reverse: ACTGCTTCCCCGTAAACAGGT | c80203.graph_c0 | Forward: AGAGATGCCGAGTTGAGAAGT  Reverse: AGCAGTAACATTCTACATTCA |
| c73572.graph_c0 | Forward: ACTGGTGACGTCGGACTTGA  Reverse: TCTGTTCAAAGAAGCTGCTGA | c75645.graph_c0 | Forward: ACGATGAGAACACAATCCAA  Reverse: TGCCATTATGAGCTCCAAGA |
| c70808.graph_c0 | Forward: TCCAAGCCATCCTCTCATGT  Reverse: TCGCAAACAAACGAAGGAAT | c77122.graph_c0 | Forward: TGCAATCTCAAGTCCGATCA  Reverse: AAGCCTCGATCAAGGGAGGT |
| c82302.graph_c2 | Forward: AGACGAGGGCTATGATCAAGT  Reverse: AAGAACCTCCTCATGCTCCT | c82361.graph_c0 | Forward: ACCCTCAGTCAGAAGACTGT  Reverse: TCTTGAGGAGCTCGCAGTAGA |
| c82572.graph_c0 | Forward: ATCGGAACAGTTGCAAAACT  Reverse: TCTGAAGTCTCCTTTGTCTT | c80561.graph_c0 | Forward: TGTGATCTCGTGATCTGGTA  Reverse: TAGTAGAAAGGTATTACGAT |
| c69542.graph_c0 | Forward: ATGACCTACTGTATCTCCAA  Reverse: TGGAGGATTTGGAAGTGATCA | c82676.graph_c0 | Forward: AAGCTCTTCAAAGAAGCGA  Reverse: TGTTACTGTAGATCTCTTCA |
| c48536.graph_c0 | Forward: TCTGTGTAGTATGAACTGCT  Reverse: AGAAATCGAGCGGTTTCGGA | c74960.graph_c0 | Forward: AAGCCCTAACTCGAAGGTTT  Reverse: TCCATGACTAGTTTCAGAGCA |
| c84481.graph_c0 | Forward: TGTCTGCCTTCAGCAGCAAA  Reverse: TCCTGATTGTGAGCTTGCGA | c61359.graph_c0 | Forward: TGCAGGGAAAACCACTGTTT  Reverse: TGACCACCAACATCCCAAACA |
| c89925.graph_c0 | Forward: AGTTCGATAACCAAAGCCTGT  Reverse: AGGTGTGATCATCTTTGCAA | c89032.graph_c0 | Forward: TGCGATCTACACGATCGCAT  Reverse: AGCAGAGCGAAGATGAACCT |
| c56621.graph_c0 | Forward: AGAAGCTGCTTTCCGTTCTT  Reverse: AAGTCTCGCCTCGAGATGGA |  |  |
