# Supplementary material for: Dynamic transcriptome profiling provides insights into rhizome enlargement in ginger (Zingiber officinale Rosc.)
Source: PLoS One. 2023 Jul 14;18(7):e0287969. doi: 10.1371/journal.pone.0287969 (PMC10348538; doi:10.1371/journal.pone.0287969)
Supplement: S4 Table — (DOCX) [file pone.0287969.s005.docx]

**S4 Table. RNA-seq data statistics of annotation results for ginger unigenes**

| **Sample Replicates** | **%≥Q30** | **Clean Reads** | **Mapped Reads** | **Mapped Ratio** |
| --- | --- | --- | --- | --- |
| S1-01 | 93.60% | 53,557,908 | 41,400,263 | 77.30% |
| S1-02 | 93.64% | 53,961,448 | 42,079,137 | 77.98% |
| S1-03 | 93.79% | 49,974,927 | 39,055,405 | 78.15% |
| S2-01 | 93.42% | 54,561,860 | 42,487,320 | 77.87% |
| S2-02 | 94.24% | 54,312,395 | 42,244,181 | 77.78% |
| S2-03 | 93.77% | 50,011,524 | 38,488,869 | 76.96% |
| S3-01 | 93.63% | 54,254,993 | 42,183,257 | 77.75% |
| S3-02 | 94.08% | 52,252,838 | 40,260,812 | 77.05% |
| S3-03 | 94.15% | 54,757,400 | 43,236,443 | 78.96% |
