# Supplementary material for: Dynamic transcriptome profiling provides insights into rhizome enlargement in ginger (Zingiber officinale Rosc.)
Source: PLoS One. 2023 Jul 14;18(7):e0287969. doi: 10.1371/journal.pone.0287969 (PMC10348538; doi:10.1371/journal.pone.0287969)
Supplement: S5 Table — (DOCX) [file pone.0287969.s006.docx]

**S5 Table. Unigenes associated with hormone biosynthesis and signaling pathway exhibiting a |Log2 FC≥1| and p≤0.05 in at least one transition**

| **Gene ID** | **FCS2/S1** | **FCS3/S2** | **Description** | **Symbol ID** | **Correlated with** |
| --- | --- | --- | --- | --- | --- |
| **Auxin** |  |  |  |  |  |
| c69273.graph_c0 | 2.54 | 0.54 | Auxin responsive protein SAUR-like | Auxin-1 |  |
| c82633.graph_c0 | -1.26 | 1.55 | Auxin responsive protein SAUR-like | Auxin-2 | ABA |
| c69847.graph_c0 | 2.70 | 0.40 | Auxin responsive protein SAUR71-like | Auxin-3 |  |
| c68464.graph_c0 | -1.73 | 1.80 | Auxin responsive protein SAUR-like | Auxin-4 | ABA |
| c76501.graph_c0 | -1.28 | 2.47 | probable auxin efflux carrier component 1c | Auxin-5 | ABA |
| c78715.graph_c0 | 0.39 | 1.87 | Auxin-induced protein PCNT107-like | Auxin-6 | JA, SL |
| **c81307.graph_c0** | **2.85** | **2.94** | **Auxin responsive protein SAUR-like** | Auxin-7 | GA |
| c44723.graph_c0 | -2.17 | 1.44 | probable auxin efflux carrier component 1b | Auxin-8 | ABA |
| c86887.graph_c0 | 0.84 | 2.46 | Auxin-induced protein 5NG4-like | Auxin-9 | IAA, JA |
| c83900.graph_c2 | -1.52 | 1.51 | Auxin responsive protein SAUR68-like | Auxin-10 | ABA |
| **c73751.graph_c0** | **3.24** | **2.95** | **Auxin-induced protein PCNT107** | Auxin-11 | GA,ZT |
| c89800.graph_c1 | 0.43 | 1.68 | Auxin-induced protein PCNT115 | Auxin-12 | JA, SL |
| c69166.graph_c0 | -1.61 | 2.98 | Auxin-regulated protein | Auxin-13 | ABA |
| c78901.graph_c1 | 2.20 | -0.74 | Auxin transporter-like protein 3 | Auxin-14 |  |
| c62365.graph_c0 | 0.28 | 2.39 | Auxin-regulated protein parC; | Auxin-15 | JA, SL |
| c62780.graph_c0 | -1.03 | 3.32 | Auxin-repressed protein 1 | Auxin-16 | ABA |
| c89679.graph_c0 | 3.17 | -2.29 | Auxin associated protein | Auxin-17 | ABA |
| c91079.graph_c0 | -1.22 | 1.75 | Auxin associated protein | Auxin-18 | ABA |
| **c82986.graph_c0** | **2.54** | **3.03** | **Auxin responsive protein SAUR-like** | Auxin-19 | GA, IAA |
| c38870.graph_c0 | -1.26 | 1.63 | Auxin-induced protein 6B | Auxin-20 | ABA |
| c69481.graph_c0 | -1.73 | 2.18 | Auxin-induced protein X10A | Auxin-21 | ABA |
| c78920.graph_c0 | 2.70 | -1.80 | IAA-amino acid hydrolase ILR1-like | Auxin-22 | ABA |
| c82518.graph_c0 | -0.28 | 2.47 | Auxin-induced protein PCNT115-like | Auxin-23 | ABA |
| c1443.graph_c0 | 3.39 | 0.87 | Auxin-induced protein 27 | Auxin-24 |  |
| c63990.graph_c1 | 0.85 | 2.94 | Auxin-induced protein 22B | Auxin-25 | IAA, JA, SL |
| c67128.graph_c0 | -2.17 | 2.44 | Auxin-induced protein PCNT103 | Auxin-26 | ABA |
| **c63780.graph_c0** | **1.84** | **3.16** | **Auxin-induced protein 22D** | Auxin-27 | GA, IAA, JA |
| c72245.graph_c0 | -1.52 | 2.51 | Auxin-induced protein PCNT115-like | Auxin-28 | ABA |
| **c54298.graph_c0** | **1.24** | **3.53** | **Auxin efflux carrier component 1-like** | Auxin-29 | IAA, JA |
| c82675.graph_c0 | 2.24 | 0.68 | probable auxin efflux carrier component 1c | Auxin-30 |  |
| c91004.graph_c0 | -1.61 | 1.98 | Auxin efflux carrier component 3 | Auxin-31 | ABA |
| c43412.graph_c0 | 2.20 | -0.74 | Auxin efflux carrier component 4-like | Auxin-32 |  |
| **Cytokinin** |  |  |  |  |  |
| c67919.graph_c0 | 2.54 | 0.19 | cytokinin-N-glucosyltransferase | CTK-1 |  |
| c79608.graph_c0 | -1.26 | 2.65 | cytokinin-N-glucosyltransferase | CTK-2 | ABA |
| c88098.graph_c0 | 2.70 | -2.52 | cytokinin riboside 5'-monophosphate phosphoribohydrolase LOG7-like | CTK-3 | ABA, BR ,SA |
| c87050.graph_c0 | -1.73 | 2.90 | Zeatin O-glucosyltransferase 3 | CTK-4 | ABA, SA |
| c73797.graph_c0 | 0.82 | 3.65 | cytokinin riboside 5'-monophosphate phosphoribohydrolase LOGL10 | CTK-5 | JA, SL |
| c73693.graph_c0 | 2.39 | 2.59 | cytokinin-N-glucosyltransferase | CTK-6 | GA |
| c75232.graph_c0 | 2.85 | -1.79 | cytokinin dehydrogenase | CTK-7 | ABA |
| c68698.graph_c0 | -2.17 | 3.42 | cytokinin-N-glucosyltransferase | CTK-8 | ABA |
| c70274.graph_c0 | 1.84 | 2.82 | cytokinin-N-glucosyltransferase | CTK-9 | GA, IAA |
| c90769.graph_c1 | -1.52 | 1.55 | cytokinin riboside 5'-monophosphate phosphoribohydrolase LOGL10 | CTK-10 | ABA |
| c86223.graph_c1 | 3.24 | 2.45 | cytokinin receptor | CTK-11 | ZT, RD |
| c71778.graph_c0 | 1.24 | 2.59 | cytokinin receptor | CTK-12 | IAA, JA |
| c74697.graph_c0 | -1.61 | 1.84 | cytokinin-N-glucosyltransferase | CTK-13 | ABA |
| c74920.graph_c0 | 3.20 | 1.46 | cytokinin receptor | CTK-14 | ZT,RD |
| **Gibberellin** |  |  |  |  |  |
| c79451.graph_c0 | 1.74 | 2.83 | gibberellin 20-oxidase | GA-1 | GA, IAA |
| c42283.graph_c0 | 2.91 | -2.14 | gibberellin-responsive protein 1 | GA-2 | ABA |
| c76856.graph_c0 | 1.55 | 3.19 | gibberellin 2-beta-dioxygenase 2-like | GA-3 | IAA, JA |
| c68340.graph_c0 | 1.97 | -1.13 | gibberellin regulated protein | GA-4 | ABA |
| c73572.graph_c0 | 2.44 | 1.67 | gibberellin receptor GID1 | GA-5 | ZT,RD |
| c75008.graph_c0 | -2.32 | 1.79 | gibberellin 2-beta-dioxygenase 8 | GA-6 | ABA |
| c63489.graph_c0 | -2.12 | 1.61 | gibberellin-regulated protein 1 | GA-7 | ABA |
| c63633.graph_c0 | 2.05 | 2.42 | gibberellin-responsive protein 1 | GA-8 | GA. IAA |
| c78790.graph_c0 | 2.13 | 2.95 | gibberellin receptor GID1 | GA-9 | GA, IAA |
| c79591.graph_c0 | 2.58 | 3.39 | gibberellin-regulated protein 14 | GA-10 | GA, IAA |
| c77741.graph_c0 | 2.14 | -2.60 | gibberellin 13-oxidase | GA-11 | ABA |
| c79045.graph_c0 | 2.46 | 3.48 | gibberellin 20-oxidase | GA-12 | GA, IAA |
| c45008.graph_c0 | 1.74 | -1.64 | gibberellin regulated protein | GA-13 | ABA |
| c70808.graph_c0 | 2.91 | 2.46 | gibberellin 3-oxidase | GA-14 | GA, ZT, RD |
| **Abscisic acid** |  |  |  |  |  |
| c72785.graph_c0 | 2.94 | -0.31 | protein responsive to abscisic acid 28 | ABA-1 |  |
| c68604.graph_c0 | -0.29 | 1.83 | ABA responsive element binding factor | ABA-2 | ABA |
| c64010.graph_c0 | -2.03 | 3.12 | abscisic acid-activated protein kinase 1 | ABA-3 | ABA |
| c75028.graph_c0 | 2.40 | -1.30 | abscisic acid-activated protein kinase 3 | ABA-4 | ABA |
| c79542.graph_c0 | -1.60 | 2.63 | abscisic acid 8'-hydroxylase | ABA-5 | ABA |
| c61618.graph_c0 | -2.03 | 1.17 | protein abscisic acid-insensitive 1 (ABI1) | ABA-6 | ABA |
| c63657.graph_c0 | -1.76 | 1.96 | protein abscisic acid-insensitive 3 (ABI3) | ABA-7 | ABA |
| **Brassinosteroid** |  |  |  |  |  |
| c82302.graph_c2 | 2.43 | 1.53 | brassinosteroid-6-oxidase 2 | BR-1 | ZT, RD |
| c91127.graph_c0 | -2.57 | 2.16 | protein brassinosteroid insensitive 1 | BR-2 | ABA |
| c73957.graph_c0 | -1.18 | 2.14 | brassinosteroid insensitive 1-associated receptor kinase 1 | BR-3 | ABA |
| c84835.graph_c0 | -1.06 | 1.93 | brassinosteroid LRR receptor kinase BRI1 | BR-4 | ABA |
| c78595.graph_c0 | 1.91 | -1.1 | brassinosteroid-signaling kinase 1 | BR-5 | ABA |
| c82572.graph_c0 | 3.15 | 2.22 | brassinosteroid resistant 1 BES1/BZR1 homolog protein 2 | BR-6 | ZT,RD |
| **Ethylene** |  |  |  |  |  |
| c86515.graph_c0 | -0.91 | 2.37 | ethylene-responsive transcription factor ERF119-like | ET-1 | ABA |
| c88061.graph_c0 | -1.21 | 3.19 | ethylene insensitive 3-like 1 protein | ET-2 | ABA |
| **Jasmonic acid** |  |  |  |  |  |
| c81152.graph_c0 | 2.17 | 2.76 | jasmonate O-methyltransferase | JA-1 | GA, IAA |
| c79546.graph_c0 | 2.07 | 3.56 | jasmonic acid-amido synthetase JAR1 | JA-2 | GA, IAA, JA |
| c63973.graph_c0 | 1.24 | 2.36 | jasmonic acid-amino synthetase | JA-3 | IAA,JA |
| **Salicylic acid** |  |  |  |  |  |
| c69542.graph_c0 | 2.24 | 1.07 | 6-methylsalicylic acid decarboxylase atA-like | SA-1 | ZT, RD |
| c78910.graph_c0 | 1.42 | -1.85 | salicylic acid 3-hydroxylase | SA-2 | ABA |
| c76379.graph_c0 | 0.19 | 2.10 | salicylic acid-binding protein | SA-3 | JA, SL |
| c69292.graph_c0 | -1.97 | 2.56 | UDP-glycosyltransferase 74G-like | SA-4 | ABA |
| c81730.graph_c0 | -2.40 | 2.83 | UDP-glycosyltransferase 74G | SA-5 | ABA |
| c71022.graph_c0 | -2.48 | 2.14 | UDP-glycosyltransferase 74F2 | SA-6 | ABA |
| **Strigolactone** |  |  |  |  |  |
| c69657.graph_c0 | 2.04 | -0.15 | probable strigolactone esterase DAD2 | SL-1 |  |
| c87176.graph_c0 | 1.41 | -0.10 | probable strigolactone esterase DAD2 | SL-2 |  |
| c71423.graph_c0 | -1.85 | 1.63 | strigolactone esterase D14 | SL-3 | ABA |
| c73727.graph_c0 | -1.43 | 1.13 | strigolactone esterase RMS3 | SL-4 | ABA |
