# Supplementary material for: Dynamic transcriptome profiling provides insights into rhizome enlargement in ginger (Zingiber officinale Rosc.)
Source: PLoS One. 2023 Jul 14;18(7):e0287969. doi: 10.1371/journal.pone.0287969 (PMC10348538; doi:10.1371/journal.pone.0287969)
Supplement: S6 Table — (DOCX) [file pone.0287969.s007.docx]

**S6 Table. Unigenes associated with transcription factors exhibiting a |Log2 FC≥1| and p≤0.05 in at least one transition**

| **Gene ID** | **FCS2/S1** | **FCS3/S2** | **Description** | **Symbol ID** | **Correlated with** |
| --- | --- | --- | --- | --- | --- |
| **MADS box** |  |  |  |  |  |
| **c88748.graph_c0** | **2.42** | **2.45** | **AGL6-like MADS box transcription factor, partial** | MB-1 | GA |
| c85594.graph_c2 | 2.09 | -0.59 | sepallata-like MADS box transcription factor 3, partial | MB-2 |  |
| c92846.graph_c0 | -1.27 | 1.46 | probable transcription factor of the MADS box family MCM1 | MB-3 | ABA |
| c80639.graph_c0 | 3.49 | -1.84 | MADS-box transcription factor 32-like | MB-4 |  |
| **c74078.graph_c0** | **1.49** | **1.51** | **MADS-box transcription factor 29-like isoform X1** | MB-5 | GA |
| c87926.graph_c0 | 1.86 | -2.01 | SEP3-like MADS-box protein | MB-6 | ABA, BR, SA |
| c74796.graph_c0 | 3.45 | -1.03 | agamous-like MADS-box protein AGL80 | MB-7 |  |
| c67174.graph_c0 | 2.53 | 0.16 | agamous-like MADS-box protein AGL65 | MB-8 |  |
| c61400.graph_c0 | 2.42 | 0.41 | MADS-box transcription factor 23-like | MB-9 |  |
| c89810.graph_c1 | -0.78 | 3.22 | Agamous-like MADS-box protein AGL80 | MB-10 | ABA, SA, SL |
| c89452.graph_c3 | -0.97 | 2.42 | MADS-box protein SOC1-like | MB-11 | ABA, SA, SL |
| **c71808.graph_c0** | **1.79** | **2.46** | **MADS-box transcription factor 3-like isoform X3** | MB-12 | GA, IAA |
| c71515.graph_c0 | -1.01 | 2.75 | MADS-box protein AGL42-like isoform X1 | MB-13 | ABA, SA, SL |
| c64351.graph_c0 | 2.95 | -0.62 | MADS-box protein SOC1-like | MB-14 |  |
| c63500.graph_c0 | 0.98 | 2.60 | SEP4 MADS-box protein | MB-15 | IAA, JA |
| c76164.graph_c0 | 3.53 | -2.30 | MADS-box transcription factor 50-like | MB-16 | ABA, BR, SA |
| c57058.graph_c0 | 2.64 | -1.54 | sepallata-like MADS box transcription factor 3, partial | MB-17 | ABA, BR, SA |
| c87139.graph_c1 | 2.72 | 0.19 | MADS-box transcription factor 26 | MB-18 |  |
| c72560.graph_c0 | 2.79 | 0.37 | PI-like MADS-box protein | MB-19 |  |
| c71638.graph_c1 | -1.01 | 2.40 | MADS-box transcription factor 51 isoform X1 | MB-20 | ABA, SA, SL |
| c70493.graph_c0 | 0.22 | 2.47 | MADS-box protein AGL17 | MB-21 | JA, SL |
| c57469.graph_c0 | -1.48 | 1.55 | SEP3b MADS-box protein | MB-22 | ABA, BR, SA |
| c67023.graph_c0 | 0.31 | 1.80 | MADS-box transcription factor 50-like | MB-23 | JA, SL |
| c5279.graph_c0 | 2.95 | 0.27 | MADS-box transcription factor 23-like isoform X1 | MB-24 |  |
| **GRAS** |  |  |  |  |  |
| c48536.graph_c0 | 3.64 | 2.94 | nodulation-signaling pathway 1 protein | GRAS-1 | GA, ZT, RD |
| c111551.graph_c0 | 3.10 | -1.87 | scarecrow-like protein 9 | GRAS-2 | ABA, BR, SA |
| c84044.graph_c0 | -1.92 | 1.44 | chitin-inducible gibberellin-responsive protein 1-like | GRAS-3 | ABA, BR, SA |
| c84548.graph_c0 | 3.09 | 0.15 | protein MONOCULM 1-like | GRAS-4 |  |
| **HB** |  |  |  |  |  |
| c19930.graph_c0 | -1.27 | 2.46 | homeobox-leucine zipper protein | HB-1 | ABA, SA |
| **c76327.graph_c0** | **3.49** | **1.51** | **homeobox-leucine zipper protein HOX4-like** | HB-2 | ZT, RD |
| c81626.graph_c1 | 3.55 | -1.95 | homeobox-leucine zipper protein HOX16 | HB-3 | ABA, BR, SA |
| c84922.graph_c0 | -2.36 | 1.68 | BEL1-like homeodomain protein 9 | HB-4 | ABA, BR, SA |
| **c84232.graph_c0** | **2.45** | **2.98** | **homeobox-DDT domain protein RLT3** | HB-5 | GA, IAA |
| c57015.graph_c0 | 0.53 | 2.74 | homeobox-leucine zipper protein ROC5-like | HB-6 | JA, SL |
| c84633.graph_c1 | 3.42 | 0.39 | homeobox-leucine zipper protein HOX12 | HB-7 |  |
| c77221.graph_c0 | -2.78 | 1.75 | homeobox-leucine zipper protein HOX12-like | HB-8 | ABA, BR, SA |
| c84162.graph_c3 | -0.97 | 2.29 | homeobox protein knotted-1-like 13 isoform X1 | HB-9 | ABA, SL |
| c76379.graph_c0 | 2.79 | 0.15 | homeobox-leucine zipper protein ROC5-like | HB-10 |  |
| c85933.graph_c0 | -2.01 | 1.63 | WUSCHEL-related homeobox 8 | HB-11 | ABA, BR, SA |
| c63293.graph_c0 | 2.95 | -2.03 | homeobox-leucine zipper protein HOX32-like | HB-12 | ABA, BR, SA |
| c83767.graph_c0 | -1.48 | 2.18 | BEL1-like homeodomain protein 9 | HB-13 | ABA, BR, SA |
| c77411.graph_c0 | -1.03 | 1.80 | homeobox-leucine zipper protein ROC5 | HB-14 | ABA, SA |
| **c85580.graph_c2** | **3.64** | **2.47** | **homeobox protein knotted-1-like 3** | HB-15 | ZT, RD |
| c71281.graph_c1 | 2.1 | -1.94 | homeobox-leucine zipper protein HOX19-like | HB-16 | ABA, BR, SA |
| c74076.graph_c0 | -1.92 | 1.87 | putative homeobox protein HOY1 | HB-17 | ABA, BR, SA |
| c36698.graph_c0 | 2.09 | 0.44 | homeobox protein knotted-1-like 3 isoform X3 | HB-18 |  |
| **c86373.graph_c0** | **1.77** | **3.16** | **homeobox-leucine zipper protein HAT4-like** | HB-19 | IAA, JA |
| c85683.graph_c1 | 0.26 | 2.51 | homeobox-leucine zipper protein HOX13-like | HB-20 | JA, SL |
| c74474.graph_c0 | 3.49 | -2.32 | homeobox-leucine zipper protein HOX9 | HB-21 | ABA, BR, SA |
| **MYB** |  |  |  |  |  |
| c78932.graph_c0 | 2.49 | -2.53 | transcription factor MYB36 | MYB-1 | ABA, BR, SA |
| c75513.graph_c0 | -1.36 | 1.68 | transcription repressor MYB6-like | MYB-2 | ABA, BR, SA |
| c39384.graph_c1 | 2.45 | 1.98 | transcriptional activator Myb-like | MYB-3 | GA, ZT, RD |
| c77838.graph_c0 | 0.37 | 2.74 | myb family transcription factor PHL8 isoform X2 | MYB-4 | JA, SL |
| c63661.graph_c0 | 2.42 | 2.39 | transcription factor MYB34-like | MYB-5 | GA |
| c80203.graph_c0 | 2.65 | 2.29 | transcription factor MYB122 | MYB-6 | GA, ZT, RD |
| c88568.graph_c0 | -1.35 | 2.7 | myb-related protein MYBAS1 isoform X2 | MYB-7 | ABA, SA |
| c85882.graph_c0 | 2.2 | -2.53 | transcription factor MYB44-like | MYB-8 | ABA, BR, SA |
| c63407.graph_c0 | -0.91 | 1.68 | myb family transcription factor EFM-like isoform X2 | MYB-9 | ABA, SA |
| c85231.graph_c0 | 0.29 | 1.98 | transcription factor MYB36-like isoform X2 | MYB-10 | JA, SL |
| c67626.graph_c0 | -2.44 | 1.41 | target of Myb protein 1-like | MYB-11 | ABA, BR, SA |
| c75645.graph_c0 | 3.53 | 2.74 | transcription factor MYB108-like | MYB-12 | ZT, RD |
| **NAC** |  |  |  |  |  |
| c90129.graph_c1 | -1.03 | 2.63 | NAC domain-containing protein 71 | NAC-1 | ABA, SA |
| c77230.graph_c0 | 0.43 | 3.18 | NAC domain-containing protein 21/22-like | NAC-2 | JA, SL |
| c83463.graph_c1 | -0.82 | 1.8 | NAC domain-containing protein 100-like | NAC-3 | ABA, SA |
| c63788.graph_c0 | 0.14 | 2.47 | NAC domain-containing protein 14-like isoform X1 | NAC-4 | JA, SL |
| c88097.graph_c0 | -1.80 | 1.87 | NAC domain-containing protein 92-like | NAC-5 | ABA, BR, SA |
| c107615.graph_c0 | 3.27 | -1.94 | NAC domain-containing protein 72 isoform X2 | NAC-6 | ABA, BR, SA |
| c80231.graph_c0 | 2.22 | 2.48 | NAC domain-containing protein 8-like | NAC-7 | GA |
| c23720.graph_c0 | -0.93 | 2.16 | NAC domain-containing protein 68-like | NAC-8 | ABA, BR, SA, SL |
| c75056.graph_c0 | 0.25 | 2.31 | NAC domain-containing protein 83-like | NAC-9 | JA, SL |
| **WRKY** |  |  |  |  |  |
| c23721.graph_c0 | -1.62 | 1.86 | putative WRKY transcription factor 43 | WRKY-1 | ABA, SA |
| c88584.graph_c0 | -2.43 | 2.38 | WRKY transcription factor 22 | WRKY-2 | ABA, BR, SA |
| c45545.graph_c0 | 2.69 | 2.47 | probable WRKY transcription factor 4 isoform X1 | WRKY-3 | GA, ZT |
| c68216.graph_c0 | 1.29 | 2.39 | probable WRKY transcription factor 4 | WRKY-4 | IAA, JA |
| c78997.graph_c0 | 0.13 | 1.29 | probable WRKY transcription factor 27 | WRKY-5 | JA, SL |
| c73811.graph_c0 | 0.09 | 1.67 | probable WRKY transcription factor 33 | WRKY-6 | JA, SL |
| c81298.graph_c0 | 0.18 | 2.11 | probable WRKY transcription factor 41 | WRKY-7 | JA, SL |
| c81133.graph_c0 | 0.25 | 3.08 | WRKY transcription factor WRKY76 | WRKY-8 | JA, SL |
| c83075.graph_c1 | 0.16 | 1.86 | probable WRKY transcription factor 21 | WRKY-9 | JA, SL |
| **bHLH** |  |  |  |  |  |
| c61211.graph_c0 | 2.29 | 2.34 | transcription factor bHLH121 | bHLH-1 | GA |
| c87421.graph_c0 | -1.16 | 2.48 | transcription factor ABA-INDUCIBLE bHLH-TYPE-like | bHLH-2 | ABA, SA |
| c77122.graph_c0 | 2.68 | 1.53 | transcription factor bHLH130 | bHLH-3 | ZT, RD |
| c87613.graph_c1 | -2.32 | 2.16 | putative transcription factor bHLH041 | bHLH-4 | ABA, BR, SA |
| c35229.graph_c1 | -0.93 | 2.14 | transcription factor bHLH13-like | bHLH-5 | ABA, SA, SL |
| c79505.graph_c0 | 2.16 | -1.1 | transcription factor bHLH69-like | bHLH-6 |  |
| c83592.graph_c0 | -2.90 | 2.22 | transcription factor bHLH144-like | bHLH-7 | ABA, BR, SA |
| c84738.graph_c1 | 1.88 | 0.46 | transcription factor bHLH54-like | bHLH-8 |  |
| c79750.graph_c0 | 2.53 | 0.31 | transcription factor bHLH128 | bHLH-9 |  |
| **bZIP** |  |  |  |  |  |
| c81227.graph_c0 | -0.96 | 2.59 | bZIP transcription factor 11-like | bZIP-1 | ABA, SA, SL |
| c81718.graph_c0 | 3.4 | 3.14 | bZIP transcription factor 27-like | bZIP-2 | GA. ZT |
| c82361.graph_c0 | 2.49 | 1.94 | bZIP transcription factor 53 | bZIP-3 | ZT, RD |
| c72216.graph_c0 | 1.84 | -2.10 | bZIP transcription factor 11-like isoform X1 | bZIP-4 | ABA, BR, SA |
| c85355.graph_c1 | -1.51 | 2.28 | bZIP transcription factor 68-like isoform X1 | bZIP-5 | ABA, SA |
| c73970.graph_c0 | 2.38 | -1.17 | bZIP transcription factor 53-like | bZIP-6 |  |
| c80561.graph_c0 | 2.12 | 1.83 | bZIP transcription factor 60 | bZIP-7 | GA, ZT, RD |
| c54678.graph_c0 | 0.28 | 3.12 | bZIP transcription factor 44-like | bZIP-8 | JA, SL |
| c88677.graph_c1 | -0.16 | 1.96 | bZIP transcription factor 27-like | bZIP-9 | ABA, SL |
| c42439.graph_c0 | 0.43 | 2.63 | bZIP transcription factor TRAB1 isoform X1 | bZIP-10 | JA, SL |
| **ARF** |  |  |  |  |  |
| c81007.graph_c1 | 3.19 | -1.30 | auxin response factor 2-like isoform X2 | ARF-1 |  |
| c62187.graph_c0 | -1.04 | 2.51 | auxin response factor 12-like isoform X1 | ARF-2 | ABA, SA, SL |
| c82676.graph_c0 | 2.28 | 1.78 | auxin response factor 17-like | ARF-3 | ZT, RD |
| c85384.graph_c0 | 2.65 | -2.4 | auxin response factor 6-like isoform X2 | ARF-4 | ABA, BR, SA |
| c74960.graph_c0 | 2.85 | 1.83 | auxin response factor 2-like | ARF-5 | ZT, RD |
| c61359.graph_c0 | 3.28 | 2.17 | auxin response factor 7 | ARF-6 | ZT, RD |
| c89032.graph_c0 | 2.38 | 1.69 | auxin response factor 17-like | ARF-7 | ZT, RD |
| c68602.graph_c0 | -1.62 | 3.36 | auxin response factor 17 | ARF-8 | ABA, SA |
| c83488.graph_c0 | 1.87 | 2.30 | auxin response factor 11 | ARF-9 | GA, IAA |
| **Aux/IAA** |  |  |  |  |  |
| c72630.graph_c0 | -1.69 | 1.59 | auxin-responsive protein IAA21-like | Aux/IAA-1 | ABA, BR, SA |
| c90323.graph_c0 | -0.04 | 1.15 | auxin-responsive protein IAA30 isoform X2 | Aux/IAA-2 |  |
| c86034.graph_c0 | 2.27 | -1.57 | auxin-responsive protein IAA25-like | Aux/IAA-3 | ABA, BR, SA |
| c72696.graph_c0 | -1.18 | 2.10 | Aux/IAA-ARF-dimerization, partial | Aux/IAA-4 | ABA, SA |
| c87739.graph_c1 | 2.82 | -2.23 | auxin-responsive protein IAA6 | Aux/IAA-5 | ABA, BR, SA |
| c88612.graph_c2 | 0.37 | 3.06 | auxin-responsive protein IAA21-like | Aux/IAA-6 | JA, SL |
| **c82667.graph_c0** | **2.01** | **2.76** | **auxin-responsive protein IAA21-like isoform X2** | Aux/IAA-7 | GA, IAA |
| c65042.graph_c0 | -1.47 | 1.56 | auxin-responsive protein IAA6 | Aux/IAA-8 | ABA, BR, SA |
| **c90322.graph_c1** | **3.42** | **2.36** | **auxin-responsive protein IAA33** | Aux/IAA-9 | ZT, RD |
| c45623.graph_c0 | 2.32 | -1.68 | auxin-responsive protein IAA16-like | Aux/IAA-10 | ABA, BR, SA |
| **c72630.graph_c1** | **2.65** | **3.17** | **auxin-responsive protein IAA30 isoform X1** | Aux/IAA-11 | GA, IAA |
| **c66906.graph_c0** | **2.54** | **2.70** | **auxin-responsive protein IAA17-like** | Aux/IAA-12 | GA |
| c83461.graph_c1 | 0.28 | 2.84 | auxin-responsive protein IAA10-like isoform X2 | Aux/IAA-13 | JA, SL |
