# Supplementary material for: Dynamic transcriptome profiling provides insights into rhizome enlargement in ginger (Zingiber officinale Rosc.)
Source: PLoS One. 2023 Jul 14;18(7):e0287969. doi: 10.1371/journal.pone.0287969 (PMC10348538; doi:10.1371/journal.pone.0287969)
Supplement: S7 Table — (DOCX) [file pone.0287969.s008.docx]

**S7 Table. Unigenes associated with cell division and expansion exhibiting a |Log2 FC≥1| and p≤0.05 in at least one transition**

| **Gene ID** | **FCS2/S1** | **FCS3/S2** | **Description** | **Symbol ID** | **Correlated with** |
| --- | --- | --- | --- | --- | --- |
| **cell division** |  |  |  |  |  |
| c74099.graph_c0 | -1.09 | 1.88 | cyclin C | CYC-1 | ABA, SA |
| c53324.graph_c0 | -1.95 | 2.43 | cyclin-D3-2-like | CYC-2 | ABA, BR, SA |
| c42709.graph_c0 | 2.43 | -1.05 | cyclin-D4-1-like | CYC-3 |  |
| c35558.graph_c0 | -1.02 | 2.72 | cyclin-A2-1-like | CYC-4 | ABA, SA, SL |
| c78505.graph_c0 | 0.98 | 2.12 | cyclin-A3-1-like | CYC-5 | IAA, JA |
| c86586.graph_c0 | 3.27 | -1.69 | cyclin-A1-4-like | CYC-6 |  |
| c84481.graph_c0 | 2.11 | 0.69 | mitotic-specific cyclin-2 | CYC-7 | RD |
| c51693.graph_c0 | 2.78 | -1.41 | cyclin-SDS-like | CYC-8 |  |
| c84059.graph_c0 | -2.16 | 2.76 | cyclin-D4-1-like | CYC-9 | ABA, BR, SA |
| c83225.graph_c0 | -1.15 | 1.93 | cyclin-A1-4-like | CYC-10 | ABA, SA |
| c74751.graph_c0 | 2.66 | -2.28 | cyclin, N-terminal domain | CYC-11 | ABA, BR, SA |
| c85673.graph_c0 | 0.93 | 2.23 | cyclin-D2-1-like | CYC-12 | IAA, JA |
| c72798.graph_c0 | -2.25 | 2.99 | cyclin-B1-2-like | CYC-13 | ABA, BR, SA |
| **c43712.graph_c0** | **1.98** | **2.64** | **cyclin-D3-2-like** | CYC-14 | GA, IAA |
| c81310.graph_c0 | 2.32 | -2.07 | Cyclin-dependent kinase E-1 | CDK-1 | ABA, BR, SA |
| c82305.graph_c1 | -3.06 | 2.54 | CDK5RAP1-like protein | CDK-2 | ABA, BR, SA |
| c89925.graph_c0 | 1.84 | 0.95 | Cyclin-dependent kinase C-2 | CDK-3 | ZT, RD |
| c10951.graph_c0 | 3.51 | -1.82 | Cyclin-dependent kinase F-4 | CDK-4 |  |
| c56621.graph_c0 | 2.45 | 0.88 | Cyclin-dependent kinase B1-1 | CDK-5 | ZT, RD |
| c81601.graph_c0 | -1.28 | 3.43 | cyclin-dependent kinase F-1-like | CDK-6 | ABA, SA, SL |
| c81352.graph_c0 | 0.74 | 2.05 | Cyclin-dependent kinase inhibitor | CDK-7 | IAA, JA |
| **c102076.graph_c0** | **1.30** | **2.72** | **Cyclin-dependent kinase A-1** | CDK-8 | IAA, JA |
| c74744.graph_c0 | -1.42 | 2.12 | Cyclin-dependent kinase G-2 | CDK-9 | ABA, SA |
| c77346.graph_c0 | -2.53 | 2.73 | Cyclin-dependent kinase inhibitor | CDK-10 | ABA, BR, SA |
| c67034.graph_c0 | 2.25 | -1.69 | cyclin-dependent kinase inhibitor 5-like | CDK-11 | ABA, BR, SA |
| c88188.graph_c0 | -2.08 | 2.41 | cyclin-dependent kinase F-4 | CDK-12 | ABA, BR, SA |
| **c90947.graph_c1** | **3.21** | **2.56** | **Cyclin-dependent kinase B2-1** | CDK-13 | GA, ZT, RD |
| c80184.graph_c0 | 2.91 | -1.5 | CDK5RAP1-like protein | CDK-14 |  |
| c89098.graph_c0 | -0.71 | 2.11 | Cyclin-dependent kinase F-4 | CDK-15 | ABA, SA, SL |
| c101334.graph_c0 | 2.51 | 0.63 | Cyclin-dependent kinase A-1 | CDK-16 |  |
| c108063.graph_c0 | -1.53 | 2.72 | Cyclin-dependent kinase E-1 | CDK-17 | ABA, SA |
| **c84494.graph_c0** | **3.32** | **2.64** | **transcription factor E2FB** | E2FB-1 | GA, ZT, RD |
| **c84948.graph_c0** | **2.99** | **1.92** | **transcription factor E2FB-like** | E2FB-2 | ZT, RD |
| **cell expansion** |  |  |  |  |  |
| c67827.graph_c0 | 2.25 | -1.11 | expansin-like B1 | EXP-1 |  |
| c80892.graph_c0 | 2.71 | -1.69 | Expansin-A1 | EXP-2 | ABA, BR, SA |
| c49020.graph_c0 | 2.98 | -1.99 | Expansin-A4 | EXP-3 | ABA, BR, SA |
| c64491.graph_c0 | -1.99 | 2.59 | Expansin-A29 | EXP-4 | ABA, BR, SA |
| **c76354.graph_c0** | **3.34** | **2.73** | **Expansin-A1** | EXP-5 | GA, ZT, RD |
| c69017.graph_c0 | 1.28 | -1.55 | Expansin-like A3 | EXP-6 | ABA, BR, SA |
| c76538.graph_c0 | 0.82 | 1.78 | Expansin-A10 | EXP-7 | IAA, JA |
| c72976.graph_c0 | -1.49 | 2.41 | Expansin-B15 | EXP-8 | ABA, SA |
| c72465.graph_c0 | -2.45 | 2.39 | Expansin-like B1 | EXP-9 | ABA, BR, SA |
| c77199.graph_c0 | 1.56 | -1.68 | expansin-B16-like | EXP-10 | ABA, BR, SA |
| c74598.graph_c0 | 2.00 | -0.85 | Cell expansion protein | EXP-11 |  |
| **c25307.graph_c0** | **2.25** | **2.47** | **expansin-B16-like** | EXP-12 | GA |
| c41665.graph_c0 | -2.3 | 2.84 | XTH30 | XTH-1 | ABA, BR, SA |
| c63898.graph_c0 | 2.98 | -1.68 | XTH31 | XTH-2 | ABA, BR, SA |
| c83677.graph_c0 | -1.99 | 3.39 | XTH30 | XTH-3 | ABA, SA |
| **c90841.graph_c0** | **3.34** | **2.19** | **XTH27** | XTH-4 | ZT, RD |
| c76046.graph_c0 | -0.98 | 2.53 | XTH32 | XTH-5 | ABA, SA |
| c45070.graph_c0 | 1.82 | -1.85 | XTH32 | XTH-6 | ABA, BR, SA |
| c72065.graph_c0 | -1.49 | 3.37 | XTH20 | XTH-7 | ABA, SA |
| c60107.graph_c0 | -2.45 | 2.08 | XTH31 | XTH-8 | ABA, BR, SA |
| c63938.graph_c0 | 2.57 | -0.92 | XTHB | XTH-9 |  |
| **c74985.graph_c0** | **3.10** | **2.21** | **XTH7** | XTH-10 | ZT, RD |
| c61787.graph_c0 | 3.54 | 0.88 | XTH5 | XTH-11 |  |
