# Supplementary material for: Dynamic transcriptome profiling provides insights into rhizome enlargement in ginger (Zingiber officinale Rosc.)
Source: PLoS One. 2023 Jul 14;18(7):e0287969. doi: 10.1371/journal.pone.0287969 (PMC10348538; doi:10.1371/journal.pone.0287969)
Supplement: S8 Table — (DOCX) [file pone.0287969.s009.docx]

**S8 Table. The correlation analysis between the content of hormones and rhizome diameter**

| Hormones  Trait | | GA3 | IAA | ABA | ZT | JA | BRs | SA | SLs |
| --- | --- | --- | --- | --- | --- | --- | --- | --- | --- |
|  |  | X1 | X2 | X3 | X4 | X5 | X6 | X7 | X8 |
| IAA | X2 | 0.992 |  |  |  |  |  |  |  |
| ABA | X3 | 0.5116 | 0.6161 |  |  |  |  |  |  |
| ZT | X4 | 0.9838 | 0.9533 | 0.3494 |  |  |  |  |  |
| JA | X5 | 0.9769 | 0.9961 | 0.6834 | 0.9228 |  |  |  |  |
| BRs | X6 | -0.479 | -0.3643 | 0.5091 | -0.6286 | -0.2804 |  |  |  |
| SA | X7 | 0.1927 | 0.3151 | 0.9417 | 0.0138 | 0.398 | 0.769 |  |  |
| SLs | X8 | 0.9154 | 0.9589 | 0.8142 | 0.8284 | 0.9803 | -0.0851 | 0.5715 |  |
| RD | Y | 0.9971 | 0.9794 | 0.4445 | 0.9946 | 0.9577 | -0.5448 | 0.1172 | 0.8819 |
